# Scalable distributed gate-model quantum computers

**DOI:** 10.1038/s41598-020-76728-5

**Published:** 2021-02-26

**Authors:** Laszlo Gyongyosi, Sandor Imre

**Affiliations:** 1 Department of Networked Systems and Services, Budapest University of Technology and Economics, Budapest, 1117 Hungary; 2 MTA-BME Information Systems Research Group, Hungarian Academy of Sciences, Budapest, 1051 Hungary

**Keywords:** Mathematics and computing, Computer science, Pure mathematics, Quantum information

## Abstract

A scalable model for a distributed quantum computation is a challenging problem due to the complexity of the problem space provided by the diversity of possible quantum systems, from small-scale quantum devices to large-scale quantum computers. Here, we define a model of scalable distributed gate-model quantum computation in near-term quantum systems of the NISQ (noisy intermediate scale quantum) technology era. We prove that the proposed architecture can maximize an objective function of a computational problem in a distributed manner. We study the impacts of decoherence on distributed objective function evaluation.

## Introduction

As the development of quantum computers evolve extensively^1–29^, the power of quantum computations has become more interpretable for efficient problem-solving. However, while experimental quantum computers are currently under development, smaller quantum devices and quantum terminals are currently available in practice. As an adequate answer to the quantum supremacy of quantum computers, the development of the quantum Internet^30–43^ has already started both in theory and experiment^32,34,36,39,44–46^, with a primary aim to provide unconditional security with advanced network services^31–34,36,38,41,44–64^. A common attribute of quantum computer architectures and the quantum Internet^30,31,52,65–119^, from an abstract theoretical point of view, are scalable distributed quantum systems^120–137^. Performing quantum computation in a distributed quantum system can also be approached as a maximization problem since a computational problem fed into the quantum system defines an objective function. The optimization of a distributed problem solving is therefore equivalent to a maximization of the objective function of a computational problem fed into the distributed quantum system (Objective function examples can be found in^5,8,9^.). A primary aim of these distributed quantum systems is therefore the maximization of an objective function in a distributed manner, via quantum CPUs in a quantum computer^1–4,14,138–141^), or by quantum terminals^39,40,45,64,125,126,128,131,133^ in a quantum Internet setting.

The problem of scalable quantum computation in a distributed quantum system is a challenge because of the complexity of the problem space provided by the diversity of possible quantum systems. The distributed quantum computational model has to include arbitrarily scaled quantum systems, from smaller quantum devices to large-scale quantum computers and the quantum Internet. As a corollary, the definition and parameterization of a scalable model for a distributed gate-model quantum computation is a hard problem, and no general solution is currently available.

Here, we study the problem of scalable quantum processing in distributed near-term quantum systems. We define a scalable distributed model of gate-model quantum computation and conceive the scaling attributes and unitaries of a distributed quantum information processing for problem-solving. The proposed scalable distributed quantum network integrates distributed quantum processing in arbitrarily scaled quantum systems.

In our context, an arbitrarily scaled quantum system can identify small, medium, or large-scale distributed quantum systems. The system model consists of an arbitrary number of quantum nodes connected by different levels of entangled connections (level of entanglement refers to the number of spanned nodes between a source and target node). The quantum system can refer to a quantum device, a quantum computer, or an arbitrary quantum Internet setting in which several quantum computers (quantum nodes) share entanglement to perform distributed quantum computations. The quantum nodes have to achieve the objective function maximization in a distributed way such that each node is allowed to apply local unitaries and connected via an arbitrary level of entanglement. In a small-scale system, the quantum nodes are connected by one-level entanglement while for a medium- or large-scale system, the level of entanglement between quantum nodes can be arbitrarily large. The local unitaries of the nodes are defined in a way that allows the distributed quantum system to implement a gate-model quantum computation in a distributed way.

We characterize a system model of a scalable distributed quantum system that allows for the performance of distributed gate-model quantum computation in a scalable manner. We define the scalable attributes of the system model and the gate parameters of the local unitaries of the quantum nodes for the objective function maximization, assuming that multipartite entanglement is utilized in the local nodes, and evaluate a cost function. The system model also assumes that the distributed quantum network evolves with time; thus, we utilize the impacts of decoherence in the distributed objective function evaluation and maximization.

Since the proposed system model is parameterizable for different physical systems, the results are applicable for distributed quantum computations in quantum computers, quantum devices, quantum networking, and the quantum Internet. Derivations focus on near-term quantum systems such as qubit-based implementations, qubit-based quantum computer architectures, and entangled network structures connected by multipartite qubit entanglement; however, the results are extendable for arbitrary dimensional quantum systems.

The novel contributions of our manuscript are as follows:

- We define a distributed quantum system to implement a scalable distributed gate-model quantum computation.
- We conceive the unitary operations of the distributed system and prove that the distributed quantum system can maximize the objective function of an arbitrary computational problem.
- We reveal the impacts of decoherence on the distributed objective function evaluation and define a suitable cost function for scalable distributed quantum computation.

This paper is organized as follows. In Sect. 2, the problem statement and system model are given. Section 3 defines the distributed quantum computational model. In Sect. 4, the scaling methods are concealed. Finally, Sect. 5 concludes the results. Supplemental information is included in the Appendix.

## Scalable distributed quantum system

### Problem statement

The issues that need to be addressed are given in Problems 1–4.

#### **Problem 1**

*Define the scaling attributes of an arbitrary distributed quantum system to resolve an arbitrary computational problem in a distributed manner.*

#### **Problem 2**

*Define the unitary operations of the distributed system that allows for the implementation of a distributed gate-model quantum computation.*

#### **Problem 3**

*Prove that the distributed quantum system can maximize the objective function of an arbitrary computational problem fed into the distributed quantum system.*

#### **Problem 4**

*Determine the impacts of decoherence on the distributed objective function evaluation, and define a suitable cost function for the distributed quantum computation.*

The resolutions to Problems 1–4 are proposed in the Theorems and Lemmas of the manuscript.

### System model

The system model of the *N* scalable distributed physical system is as follows. In $N=\left( V,S\right)$, the $\left| V\right|$ quantum nodes are connected via $\left| S\right|$, *l*-level entangled connections (An entangled connection between the quantum nodes refers to a shared multipartite entanglement. Between two nodes, *x* and *y*, the entangled connection identifies a bipartite quantum entanglement. For a qubit setting, the $d=2$ dimensional twopartite maximally entangled states are the so-called Bell states; here one can assume the use of the $| \beta _{00} \rangle$ state, ${\left| \beta _{00} \right\rangle } ={\textstyle \frac{1}{\sqrt{2} }} \left( {\left| 00 \right\rangle } +{\left| 11 \right\rangle } \right)$ in the system model.), where *V* is a set of quantum nodes and *S* is a set of entangled connections. For an *l*-level entangled connection, the $d{\left( x,y\right) }_{\text {L}_l}$ hop distance in *N* is

1 $$\begin{aligned} d{\left( x,y\right) }_{\text {L}_l}=2^{l-1}, \end{aligned}$$

with $d{\left( x,y\right) }_{\text {L}_l}-1$ intermediate nodes (The level *l* of an entangled connection assumes that each entanglement level doubles the hop-distance between *x* and *y*, which is a general model in quantum networking. It is also used in the so-called doubling architecture of entanglement distribution, in which the entanglement levels are increased via entanglement swapping^39,41^. Note, that *l* can model any hop-distance between the nodes.) between the nodes *x* and *y*. Thus, $l=1$ refers to a direct connection between two quantum nodes *x* and *y* without intermediate quantum nodes in the distributed system *N*.

#### **Proposition 1**

*(Distributed quantum system for a scalable distributed gate-model quantum computation). An arbitrary distributed quantum system N is scalable via the entanglement level of entangled connections, by the gate parameters of the local unitaries of the quantum nodes, and by local measurements in the nodes. The N distributed quantum system can implement a scalable gate-model quantum computation in a distributed manner*.

#### *Proof*

The level *l* of the entangled connections between the nodes depends on the physical size and topology of *N*. Without loss of generality, for an *s* small-scale distributed quantum system (quantum device, quantum terminal, smaller quantum computer),

2 $$\begin{aligned} l =1, \end{aligned}$$

while for an *m* medium, or *l* large-scale distributed quantum system (medium or large-scale quantum computer, quantum repeater network, arbitrary quantum communication network, quantum Internet),

3 $$\begin{aligned} l \ge 1. \end{aligned}$$

A $${\mathcal {P}}\left( A\rightarrow B\right) $$ computational path of *N* is modeled as a set $$V=\left\{ V_{1} ,\ldots ,V_{L} \right\} $$ of *L* quantum nodes, with a set $$S=\left\{ E_{1} ,\ldots ,E_{L-1} \right\} $$ of $$L-1$$ entangled connections between the nodes, where $$E_{j} $$ identifies an entangled connection between *d*-dimensional quantum states *j* and *k* in nodes $$V_{x} $$ and $$V_{y} $$ . Focusing on near-term distributed quantum systems, we use $$d=2$$; thus, *j* and *k* refer to qubits throughout the manuscript. The aim of the $${\mathcal {P}}\left( A\rightarrow B\right) $$ computational path is to maximize a particular objective function $$C_{{\mathcal {P}}\left( A\rightarrow B\right) } $$ of an arbitrary computational problem in a distributed manner using the nodes and entangled connections of the path.

The allowed operations for a node pair $$V_{xy} =\left\{ V_{x} ,V_{y} \right\} $$ with a shared *l*-level entangled connection $$E_{j} $$, $$j=1,\ldots ,L-1$$ are defined as follows.

A scalable gate-model quantum computation can be set up in *N* by allowing the local nodes to perform local unitaries using the Pauli $\sigma _{x}$ and $\sigma _{z}$ operators. The local unitaries scaled by the gate parameters, in the following manner.

A node pair $\left\{ V_{x} ,V_{y} \right\}$ is allowed perform a local single-qubit unitaries^12,14^

4 $$\begin{aligned} U\left( X_{j} ,\beta _{j} \right) =\exp \left( -i\beta _{j} X_{j} \right) , \end{aligned}$$

where $\beta _{j} \in \left[ 0,\pi \right]$ is the gate parameter of the unitary, while *X* is the Pauli $\sigma _{x}$ operator, and

5 $$\begin{aligned} U\left( X_{k} ,\beta _{k} \right) =\exp \left( -i\beta _{k} X_{k} \right) , \end{aligned}$$

on qubits *j* and *k* in nodes $$V_{x} $$ and $$V_{y} $$, $$\beta _{k} \in \left[ 0,\pi \right] $$.

The node pair is also allowed to realize a distributed unitary

6 $$\begin{aligned} U\left( Z_{j} Z_{k} ,\gamma _{jk} \right) =U\left( Z_{j} Z_{k} ,\gamma _{j} \right) U\left( Z_{j} Z_{k} ,\gamma _{k} \right) = \exp \left( -i\left( {{\gamma }_{j}} \right) {{Z}_{j}}{{Z}_{k}} \right) \exp \left( -i\left( {{\gamma }_{k}} \right) {{Z}_{j}}{{Z}_{k}} \right) \end{aligned}$$

on qubits *j* and *k* using the *l*-level entangled connection $E_{j} =\left\langle jk\right\rangle$, where $\gamma _{jk} \in \left[ 0,2\pi \right]$ is the gate parameter of the distributed unitary^12,14^, defined as

7 $$\begin{aligned} \gamma _{jk} =\gamma _{j} +\gamma _{k} , \end{aligned}$$

where $\gamma _{j} ,\gamma _{k} \in \left[ 0,\pi \right]$ are the local gate parameters applied on qubits *j* and *k*, *Z* is the Pauli $\sigma _{z}$ operator, while

8 $$\begin{aligned} \begin{aligned}    & U\left( {{Z}_{j}}{{Z}_{k}},{{\gamma }_{jk}} \right) \\&\quad =U\left( {{Z}_{j}}{{Z}_{k}},{{\gamma }_{j}} \right) U\left( {{Z}_{j}}{{Z}_{k}},{{\gamma }_{k}} \right) \\&\quad =\exp \left( -i\left( {{\gamma }_{j}} \right) {{Z}_{j}}{{Z}_{k}} \right) \exp \left( -i\left( {{\gamma }_{k}} \right) {{Z}_{j}}{{Z}_{k}} \right) \\&\quad =\left( \cos \left( {{\gamma }_{j}} \right) I-i\sin \left( {{\gamma }_{j}} \right) {{Z}_{j}}{{Z}_{k}} \right) \left( \cos \left( {{\gamma }_{k}} \right) I-i\sin \left( {{\gamma }_{k}} \right) {{Z}_{j}}{{Z}_{k}} \right) \\&\quad =\cos \left( {{\gamma }_{j}} \right) \cos \left( {{\gamma }_{k}} \right) I-i\cos \left( {{\gamma }_{j}} \right) \sin \left( {{\gamma }_{k}} \right) {{Z}_{j}}{{Z}_{k}}-i\cos \left( {{\gamma }_{k}} \right) \sin \left( {{\gamma }_{j}} \right) {{Z}_{j}}{{Z}_{k}}-\sin \left( {{\gamma }_{j}} \right) \sin \left( {{\gamma }_{k}} \right) {{Z}_{j}}{{Z}_{k}}{{Z}_{j}}{{Z}_{k}} \\&\quad =\tfrac{1}{2}\left( \cos \left( {{\gamma }_{j}}-{{\gamma }_{k}} \right) +\cos \left( {{\gamma }_{j}}+{{\gamma }_{k}} \right) \right) I-i\left( \tfrac{1}{2}\left( \sin \left( {{\gamma }_{j}}+{{\gamma }_{k}} \right) -\sin \left( {{\gamma }_{j}}-{{\gamma }_{k}} \right) \right) \right) {{Z}_{j}}{{Z}_{k}} \\&\qquad-i\left( \tfrac{1}{2}\left( \sin \left( {{\gamma }_{j}}+{{\gamma }_{k}} \right) -\sin \left( {{\gamma }_{k}}-{{\gamma }_{j}} \right) \right) \right) {{Z}_{j}}{{Z}_{k}}-\tfrac{1}{2}\left( \cos \left( {{\gamma }_{j}}-{{\gamma }_{k}} \right) -\cos \left( {{\gamma }_{j}}+{{\gamma }_{k}} \right) \right) I \\&\quad =\cos \left( {{\gamma }_{j}}+{{\gamma }_{k}} \right) I-2i\left( \tfrac{1}{2}\left( \sin \left( {{\gamma }_{j}}+{{\gamma }_{k}} \right) -\sin \left( {{\gamma }_{k}}-{{\gamma }_{j}} \right) \right) \right) {{Z}_{j}}{{Z}_{k}}. \end{aligned} \end{aligned}$$

Thus, setting

9 $$\begin{aligned} \gamma _{j} =\gamma _{k} ={\textstyle \frac{1}{2}} \gamma _{jk} , \end{aligned}$$

the result in (8) can be evaluated as

10 $$\begin{aligned} \begin{aligned} U\left( {{Z}_{j}}{{Z}_{k}},{{\gamma }_{jk}} \right)&=U\left( {{Z}_{j}}{{Z}_{k}},{{\gamma }_{j}} \right) U\left( {{Z}_{j}}{{Z}_{k}},{{\gamma }_{k}} \right) \\&=\cos \left( {{\gamma }_{jk}} \right) I-i\sin \left( {{{{\gamma }'_{jk}}}} \right) {{Z}_{j}}{{Z}_{k}} \\&=\exp \left( -i\left( {{\gamma }_{jk}} \right) {{Z}_{j}}{{Z}_{k}} \right) . \end{aligned} \end{aligned}$$

A node $$V_{x} $$ can also apply an $$U_{x}^{C} $$ local coupling unitary to connect qubits *i* and *j* from entangled connections $$\left\langle \left( i-1\right) \left( i\right) \right\rangle $$ and $$\left\langle jk\right\rangle $$ in $$V_{x} $$, as

11 $$\begin{aligned} U_{x}^{C} =\exp \left( -itH^{\left( i,j\right) } \right) \end{aligned}$$

where $$H^{\left( i,j\right) }$$ is a Hamiltonian, and also in $$V_{y} $$ on the qubits *k* and $$k+1$$ of entangled connections $$\left\langle jk\right\rangle $$ and $$\left\langle \left( k+1\right) \left( k+2\right) \right\rangle $$, as

12 $$\begin{aligned} U_{y}^{C} =\exp \left( -itH^{\left( k,k+1\right) } \right) \end{aligned}$$

where $H^{\left( k,k+1\right) }$ is a Hamiltonian, to connect qubits *k* and $k+1$, and remote entangled connections.

Therefore, the $$U_{xy} $$ unitary associated to a given node pair $$\left\{ V_{x} ,V_{y} \right\} $$ connected by an *l*-level entanglement $$E_{j} $$ in the distributed quantum system *N* is defined as

13 $$\begin{aligned} \begin{aligned} {{U}_{xy}}&={{U}_{x}}{{U}_{y}} \\&=U\left( {{B}_{j}},{{\beta }_{j}} \right) U\left( {{Z}_{j}}{{Z}_{k}},{{\gamma }_{j}} \right) U\left( {{B}_{k}},{{\beta }_{k}} \right) U\left( {{Z}_{j}}{{Z}_{k}},{{\gamma }_{k}} \right) \\&=U\left( {{X}_{j}},{{\beta }_{j}} \right) U\left( {{X}_{k}},{{\beta }_{k}} \right) U\left( {{Z}_{j}}{{Z}_{k}},{{\gamma }_{jk}} \right) U_{x}^{C}U_{y}^{C} \\&=\exp \left( -i{{\beta }_{j}}{{X}_{j}} \right) \exp \left( -i{{\beta }_{k}}{{X}_{k}} \right) \exp \left( -i{{\gamma }_{jk}}{{Z}_{j}}{{Z}_{k}} \right) \exp \left( -it{{H}^{\left( i,j \right) }} \right) \exp \left( -it{{H}^{\left( k,k+1 \right) }} \right) , \end{aligned} \end{aligned}$$

where $$U_{x} $$ is the unitary of a node $$V_{x} $$, $$x=1,\ldots ,L$$, defined as

14 $$\begin{aligned} U_{x} =U\left( X_{j} ,\beta _{j} \right) U\left( Z_{j} Z_{k} ,\gamma _{j} \right) , \end{aligned}$$

while $$U_{y} $$ is the unitary of its neighbor node $$V_{y} $$, as

15 $$\begin{aligned} U_{y} =U\left( X_{k} ,\beta _{k} \right) U\left( Z_{j} Z_{k} ,\gamma _{k} \right) . \end{aligned}$$

Since unitaries (14) and (15) allows us to realize a gate-model quantum computation^14,29^, it follows that the $\left\{ V_{x} ,V_{y} \right\}$ node pairs of the distributed quantum system *N* can implement quantum computation using their entangled connections in a distributed manner. $\square$

### Methods

#### **Proposition 2**

*To model multipartite entanglement in a particular node* $$V_{x} $$, *qubit* *j* *has entangled connection with* *k* *to formulate* $$\left\langle jk\right\rangle $$, *and also with* $$\Gamma _{j} $$ *remote qubits*, $$n_{1} ,\ldots ,n_{\Gamma _{j} } $$, *which are not neighbors of qubit k (These* $$\Gamma _{j} $$ *qubits have no connections with qubit k.)*. *The total number of qubits that are neighbor of j* *but not neighbor of k is* $$\Gamma _{j} +1$$.

#### *Proof*

Each entangled connection $$E_{j} $$ has a contribution $$\zeta _{E_{j} } $$ to an $$F_{{\mathcal {P}}\left( A\rightarrow B\right) } $$ target function of a computational path $${\mathcal {P}}\left( A\rightarrow B\right) $$ (will be proven in Sect. 3)

16 $$\begin{aligned} \begin{aligned} {{F}_{\mathcal {P}\left( A\rightarrow B \right) }}&=\underset{\forall \theta }{\mathop {\max }}\,\left\langle {{\varphi }^{*}} | \left. {{C}_{\mathcal {P}\left( A\rightarrow B \right) }} \right| {{\varphi }^{*}} \right\rangle \\&=\tfrac{1}{2}\sum \nolimits _{j=1}^{L-1}{{{\zeta }_{{{E}_{j}}}}}, \end{aligned} \end{aligned}$$

where ${\left| \varphi ^{*} \right\rangle }$ is the output state of ${\mathcal {P}}\left( A\rightarrow B\right)$, defined as

17 $$\begin{aligned} {\left| \varphi ^{*} \right\rangle } =U_{{\mathcal {P}}\left( A\rightarrow B\right) } {\left| + \right\rangle } , \end{aligned}$$

where ${\left| + \right\rangle } ={\textstyle \frac{1}{\sqrt{2} }} \left( {\left| 0 \right\rangle } +{\left| 1 \right\rangle } \right)$, while $U_{{\mathcal {P}}\left( A\rightarrow B\right) }$ is defined as a unitary sequence associated to ${\mathcal {P}}\left( A\rightarrow B\right)$, as

18 $$\begin{aligned} \begin{aligned} {{U}_{\mathcal {P}\left( A\rightarrow B \right) }}=&{{U}_{L}}{{U}_{L-1}}\ldots {{U}_{1}} \\ =&U\left( {{X}_{L}},{{\beta }_{L}} \right) U\left( {{X}_{L-1}},{{\beta }_{L-1}} \right) U\left( {{Z}_{L-1}}{{Z}_{L}},{{\gamma }_{L-1,L}} \right) U\left( {{Z}_{L-1}}{{Z}_{L-2}},{{\gamma }_{L-1,L-2}} \right) \\&\ldots U\left( {{X}_{2}},{{\beta }_{2}} \right) U\left( {{X}_{1}},{{\beta }_{1}} \right) U\left( {{Z}_{1}}{{Z}_{2}},{{\gamma }_{12}} \right) \\ =&\prod \limits _{j\in \mathcal {P}\left( A\rightarrow B \right) }{U\left( {{X}_{j}},{{\beta }_{j}} \right) \prod \limits _{\left\langle jk \right\rangle \in \mathcal {P}\left( A\rightarrow B \right) }{U\left( {{Z}_{j}}{{Z}_{k}},{{\gamma }_{jk}} \right) ,}} \end{aligned} \end{aligned}$$

where $$\left\langle jk\right\rangle \in {\mathcal {P}}\left( A\rightarrow B\right) $$ refers to an $$E_{j} $$ entangled connection between qubits *j* and *k* on the computational path $${\mathcal {P}}\left( A\rightarrow B\right) $$.

The *n*-qubit length input system ${\left| s \right\rangle }$ of the distributed system *N*, is defined as a product of $\sigma _{x}$ eigenstates^12,14^, as

19 $$\begin{aligned} {\left| s \right\rangle } ={\left| + \right\rangle } _{1} {\left| + \right\rangle } _{2} \ldots {\left| + \right\rangle } _{n} ={\left| + \right\rangle } ^{\otimes n} ={\textstyle \frac{1}{\sqrt{2^{n} } }} \sum _{z}{\left| z \right\rangle } , \end{aligned}$$

where ${\left| z \right\rangle }$ is a computational basis state, *z* is an *n*-length string,

20 $$\begin{aligned} z=z_{1} z_{2} \ldots z_{n} , \end{aligned}$$

where $$z_{i} $$ identifies an *i*-th bit, $$z_{i} \in \left\{ -1,1\right\} $$, and $${\left| + \right\rangle } _{i} $$ is the input system of an *i*-th computational path (As *N* is a quantum computer system or a quantum device with quantum registers, then $${\left| s \right\rangle } $$ refers to a quantum register in the superposition of *n* qubits, while a given node $$A_{i} $$ identifies the *i*-th source qubit, $${\left| + \right\rangle } _{i} $$, of the *n*-length quantum register. In the current system model, the input system fed into the distributed system can also refer to a quantum register, physically not distributed between distant parties.) $${\mathcal {P}}\left( A_{i} \rightarrow B_{i} \right) $$.

The nodes of the distributed system also can perform $M\left[ m_{b} \right]$ local measurements in a base $m_{b} \in \left\{ m_{0} ,m_{1} \right\}$ (see (35), (36)) to realize an ${{\mathcal L}}_{U}$ upload^76,142^ and an ${{\mathcal L}}_{D}$ download^76,143^ procedure. The ${{\mathcal L}}_{U}$ upload procedure is an information delocalization method^76,142^, in which a source system is uploaded by a source node onto the network state formulated by the entangled connections of the intermediate nodes of the distributed system. The ${{\mathcal L}}_{D}$ download procedure is an information localization procedure^76,143^, in which the uploaded and transformed information (transformed by the local unitaries of the quantum nodes in our setting) is localized into a particular target node from the network state of intermediate nodes. Since the distributed quantum system evolves with time, the timing of a local measurement also represents a scalable attribute in the distributed system (see (27) and (37)). $\square$

#### **Proposition 3**

*In a source node* $$A_{i} $$, *the* $${{\mathcal L}}_{U} \left( {\left| + \right\rangle } _{i} \right) $$ *uploading is realized by a* $${{\mathcal M}}_{B} $$ *Bell measurement*^76^ *applied on input system* $${\left| + \right\rangle } _{i} $$ *and the first particle of chain* $${\left| \Phi \right\rangle } _{i} $$ *that identifies the* $${\left| \Phi \right\rangle } _{i}$$ *network state of computational path* $${\mathcal {P}}\left( A_{i} \rightarrow B_{i} \right) $$.

#### *Proof*

The ${\left| \Phi \right\rangle } _{i}$ network state is defined as

21 $$\begin{aligned} {\left| \Phi \right\rangle } _{i} =U(\vec {\theta }_{i}){\textstyle \frac{1}{\sqrt{2} }} \left( {\left| 0 \right\rangle } _{aux} \left( {\left| 0 \right\rangle } \right) _{2}^{2\left( L-1\right) } +{\left| 1 \right\rangle } _{aux} \left( {\left| 1 \right\rangle } \right) _{2}^{2\left( L-1\right) } \right) , \end{aligned}$$

where sub-index 1 identifies the first particle of ${\left| \Phi \right\rangle } _{i}$ of ${\mathcal {P}}\left( A_{i} \rightarrow B_{i} \right)$ maximally entangled with the remaining $2\left( L-1\right)$ qubits of the chain of ${\mathcal {P}}\left( A_{i} \rightarrow B_{i} \right)$.

The ${{\mathcal L}}_{U} \left( {\left| + \right\rangle } _{i} \right)$ uploading process results in

22 $$\begin{aligned} \begin{aligned} {{\mathcal {L}}_{U}}\left( {{\alpha }_{i}}\left| 0 \right\rangle +{{\beta }_{i}}\left| 1 \right\rangle \right)&=U\left( {{{\vec {\theta }}}_{i}} \right) \left( {{\alpha }_{i}}\left( \left| 0 \right\rangle \right) _{2}^{2\left( L-1 \right) }+{{\beta }_{i}}\left( \left| 1 \right\rangle \right) _{2}^{2\left( L-1 \right) } \right) \\&=U\left( {{{\vec {\theta }}}_{i}} \right) \tfrac{1}{\sqrt{2}}\left( \left( \left| 0 \right\rangle \right) _{2}^{2\left( L-1 \right) }+\left( \left| 1 \right\rangle \right) _{2}^{2\left( L-1 \right) } \right) , \end{aligned} \end{aligned}$$

where $$\alpha _{i} {\left| 0 \right\rangle } +\beta _{i} {\left| 1 \right\rangle } ={\textstyle \frac{1}{\sqrt{2} }} \left( {\left| 0 \right\rangle } +{\left| 1 \right\rangle } \right) ={\left| + \right\rangle } _{i} $$.

Applying ${{\mathcal L}}_{U} \left( {\left| + \right\rangle } _{i} \right)$ for $i=1,\ldots ,n$, uploads the input system ${\left| s \right\rangle }$ in a distributed manner, as

23 $$\begin{aligned} {{\mathcal L}}_{U} \left( {\left| s \right\rangle } \right) ={{\mathcal L}}_{U} \left( {\left| + \right\rangle } _{1} {\left| + \right\rangle } _{2} \ldots {\left| + \right\rangle } _{n} \right) , \end{aligned}$$

to the ${\left| \Phi \right\rangle } _{1}^{n}$ distributed network state formulated via *n* computational paths ${\mathcal {P}}\left( A_{1} \rightarrow B_{1} \right) ,\ldots ,{\mathcal {P}}\left( A_{n} \rightarrow B_{n} \right)$, as

24 $$\begin{aligned} \begin{aligned} \left| \Phi \right\rangle _{1}^{n}&=U\left( N \right) \tfrac{1}{\sqrt{2}}\left( \left( \left| 00 \right\rangle \right) _{1}^{n2\left( L-1 \right) }+\left( \left| 11 \right\rangle \right) _{1}^{n2\left( L-1 \right) } \right) \\&=U\left( N \right) \tfrac{1}{\sqrt{2}}\left( \left| 0 \right\rangle _{1}^{n}\left( \left| 0 \right\rangle \right) _{n+1}^{n2\left( L-1 \right) }+\left| 1 \right\rangle _{1}^{n}\left( \left| 1 \right\rangle \right) _{n+1}^{n2\left( L-1 \right) } \right) , \end{aligned} \end{aligned}$$

while indices $1,\ldots ,n$ identify the auxiliary systems used for the uploading procedure in the *n* source nodes^76,142^, while $U\left( N\right)$ the unitary of *N* is defined as,

$$\begin{aligned} U\left( N\right) =\prod _{j\in N}U\left( X_{j} ,\beta _{j} \right) \prod _{\left\langle jk\right\rangle \in N}U\left( Z_{j} Z_{k} ,\gamma _{jk} \right) =U\left({\vec{\theta}}_{n} \right) U\left({\vec{\theta}}_{n-1} \right) \ldots U\left({\vec{\theta}}_{1} \right) , \end{aligned}$$

where $U(\vec {\theta }_{i})$ refer to the unitary associated to an *i*-th path ${\mathcal {P}}\left( A_{i} \rightarrow B_{i} \right)$, $i=1,\ldots ,n$, defined as a unitary sequence of *L* unitaries,

25 $$\begin{aligned} U\left({\vec{\theta}}_{i}\right) =U_{i,L} U_{i,L-1} \ldots U_{i,1} , \end{aligned}$$

where $$U_{i,x} $$ identifies the unitary of a given node $$V_{x} $$ of $${\mathcal {P}}\left( A_{i} \rightarrow B_{i} \right) $$, as

26 $$\begin{aligned} U_{i,1} =U\left( \beta _{i,1} ,X_{i,1} \right) U\left( \gamma _{i,1} ,Z_{i,1} \right) , \end{aligned}$$

and $$U(\vec {\theta }_{i})$$ is a unitary sequence of 2*L* unitaries implemented via *L* nodes $$V_{x} $$, $$x=1,\ldots ,L$$, in $${\mathcal {P}}\left( A_{i} \rightarrow B_{i} \right) $$.

The ${{\mathcal L}}_{U} \left( {\left| s \right\rangle } \right)$ operation therefore results in

27 $$\begin{aligned} {{\mathcal L}}_{U} \left( {\left| s \right\rangle } \right) =U\left( N\right) {\textstyle \frac{1}{\sqrt{2^{n} } }} \left( \left( {\left| 0 \right\rangle } \right) _{n+1}^{n2\left( L-1\right) } +\left( {\left| 1 \right\rangle } \right) _{n+1}^{n2\left( L-1\right) } \right) , \end{aligned}$$

that yields the output system of *N*

28 $$\begin{aligned} {\left| \phi ^{*} \right\rangle } =U\left( N\right) {\left| s \right\rangle } \end{aligned}$$

distributed between the *n* receiver nodes $B_{1} ,\ldots ,B_{n}$. Thus. the outputs of the *n* paths, $U\left( N\right) {\left| s \right\rangle }$ can be localized onto the *n* receivers in the downloading procedure^76,143^.

To verify (22) and (27), we recall the formalisms of^144,145^. The input system $${\left| + \right\rangle } _{i} $$ of a given node $$A_{i} $$ can be rewritten as

29 $$\begin{aligned} {\left| + \right\rangle } _{i} =\lambda _{0} {\left| 0 \right\rangle } +\lambda _{1} {\left| 1 \right\rangle } , \end{aligned}$$

and let ${\left| \Phi \right\rangle } _{i}$ be as given in (21), then

30 $$\begin{aligned} {\left| \Phi \right\rangle } _{i} {\left| + \right\rangle } _{i} =\sum _{k,s=0,1}\lambda _{k} U(\vec {\theta }_{i}){\textstyle \frac{1}{\sqrt{2} }} \left( \left( M\left[ m_{s} \right] {\left| L \right\rangle } \right) _{2}^{2\left( L-1\right) } \right) {\left| m_{s} \right\rangle } _{1} {\left| k \right\rangle } _{0} , \end{aligned}$$

where indices 0 and 1 identify the input system ${\left| + \right\rangle } _{i}$ and the first qubit of the first EPR pair of chain ${\left| \Phi \right\rangle } _{i}$ that serves as an ${\left| aux \right\rangle }$ auxiliary qubit system, ${{\mathcal H}}_{aux} ={\mathbb {C}}^{2}$, maximally entangled with the $2\left( L-1\right)$-qubit length system $\left( {\left| L \right\rangle } \right) _{2}^{2\left( L-1\right) }$, ${{\mathcal H}}_{L} ={\mathbb {C}}^{2^{\otimes 2\left( L-1\right) } }$, formulating orthogonal states as $\left( {\left| L \right\rangle } \right) _{2}^{2\left( L-1\right) } =\left\{ {\left| 0 \right\rangle } _{2}^{2\left( L-1\right) } ,{\left| 1 \right\rangle } _{2}^{2\left( L-1\right) } \right\}$, while $\left\{ {\left| m_{s} \right\rangle } \right\}$ is an orthogonal basis^76,144,145^.

Then, in node $$A_{i} $$ an $${{\mathcal M}}_{B} $$ Bell measurement is applied on subsystems 0 and 1, that yields a projection onto

31 $$\begin{aligned} {{\mathcal M}}_{B} \left( A_{i} \right) :{\left| B_{1} \right\rangle } _{10} =\sum _{j}{\left| m_{j} \right\rangle } _{1} {\left| j \right\rangle } _{0} , \end{aligned}$$

while the ${\left| \Phi ' \right\rangle } _{i}$ post-measurement network state is evaluated as

32 $$\begin{aligned} \begin{aligned} {{\left| {{\Phi }'} \right\rangle }_{i}}&=\sum \limits _{j}{U\left( {{{\vec {\theta }}}_{i}} \right) \left( {{\lambda }_{j}}M\left[ {{m}_{j}} \right] \left( \left| L \right\rangle \right) _{2}^{2\left( L-1 \right) } \right) } \\&=U\left( {{{\vec {\theta }}}_{i}} \right) \left( {{\lambda }_{0}}\left( \left| 0 \right\rangle \right) _{2}^{2\left( L-1 \right) }+{{\lambda }_{0}}\left( \left| 1 \right\rangle \right) _{2}^{2\left( L-1 \right) } \right) \\&=U\left( {{{\vec {\theta }}}_{i}} \right) \tfrac{1}{\sqrt{2}}\left( \left( \left| 0 \right\rangle \right) _{2}^{2\left( L-1 \right) }+\left( \left| 1 \right\rangle \right) _{2}^{2\left( L-1 \right) } \right) \\&={{\mathcal {L}}_{U}}\left( \tfrac{1}{\sqrt{2}}\left( \left| 0 \right\rangle +\left| 1 \right\rangle \right) \right) , \end{aligned} \end{aligned}$$

which coincidences with (22).

Extending the derivations to *n* computational paths such that the paths realize the *n*-qubit unitary $$U\left( N\right) $$, each $$A_{i} $$ apply a Bell measurement $${{\mathcal M}}_{B} \left( A_{i} \right) $$, thus the post-measurement network state $${\left| \Phi ' \right\rangle } _{1}^{n} $$ is as

33 $$\begin{aligned} \begin{aligned} \left| {{\Phi }'} \right\rangle _{1}^{n}&=U\left( N \right) \sum \limits _{i,j}{\left( {{\lambda }_{i,j}}M\left[ {{m}_{i,j}} \right] \left( \left| L \right\rangle \right) _{n+1}^{n2\left( L-1 \right) } \right) } \\&=U\left( N \right) \left( {{\lambda }_{1,0}}{{\lambda }_{2,0}}\ldots {{\lambda }_{n,0}}\left( \left| 0 \right\rangle \right) _{n+1}^{n2\left( L-1 \right) }+{{\lambda }_{1,1}}{{\lambda }_{2,1}}\ldots {{\lambda }_{n,1}}\left( \left| 1 \right\rangle \right) _{n+1}^{n2\left( L-1 \right) } \right) \\&=U\left( N \right) \left( \lambda _{0}^{n}\left( \left| 0 \right\rangle \right) _{n+1}^{n2\left( L-1 \right) }+\lambda _{1}^{n}\left( \left| 1 \right\rangle \right) _{n+1}^{n2\left( L-1 \right) } \right) \\&=U\left( N \right) \tfrac{1}{\sqrt{{{2}^{n}}}}\left( \left( \left| 0 \right\rangle \right) _{n+1}^{n2\left( L-1 \right) }+\left( \left| 1 \right\rangle \right) _{n+1}^{n2\left( L-1 \right) } \right) \\&={{\mathcal {L}}_{U}}\left( {{\left| + \right\rangle }_{1}}{{\left| + \right\rangle }_{2}}\ldots {{\left| + \right\rangle }_{n}} \right) , \end{aligned} \end{aligned}$$

where $\left( {\left| L \right\rangle } \right) _{n+1}^{n2\left( L-1\right) } =\left\{ {\left| 0 \right\rangle } _{n+1}^{n2\left( L-1\right) } ,{\left| 1 \right\rangle } _{n+1}^{n2\left( L-1\right) } \right\}$ defined on ${{\mathcal H}}_{L} ={\mathbb {C}}^{2^{\otimes n2\left( L-1\right) } }$ since the entangled network structure of the distributed system is formulated via $n2\left( L-1\right)$ entangled states over the *n* computational paths, while *n* auxiliary qubit systems, ${\left| aux \right\rangle } _{1} {\left| aux \right\rangle } _{2} \ldots {\left| aux \right\rangle } _{n}$, are measured via the Bell measurements in the *n* source nodes, ${{\mathcal H}}_{aux_{1\ldots n} } ={\mathbb {C}}^{2^{\otimes n} }$, that confirms the result in (27).

The $${{\mathcal L}}_{D} $$ downloading process^76,143^ for receiver node $$B_{i} $$ results in

34 $$\begin{aligned} {{\mathcal L}}_{D} \left( U({\vec{\theta}}_{i}){\textstyle \frac{1}{\sqrt{2} }} \left( \left( {\left| 0 \right\rangle } \right) _{2}^{2\left( L-1\right) } +\left( {\left| 1 \right\rangle } \right) _{2}^{2\left( L-1\right) } \right) \right) =U({\vec{\theta}}_{i}){\left| + \right\rangle } _{i} . \end{aligned}$$

To obtain (34) in $$B_{i} $$, $$M\left[ m_{b} \right] $$ local measurements in a $$m_{b} $$ suitable basis are applied on the remaining $$2\left( L-1\right) -1$$ qubits in the $$L-1$$ nodes of $${\mathcal {P}}\left( A_{i} \rightarrow B_{i} \right) $$ between $$A_{i} $$ and $$B_{i} $$. Recalling the formalisms of^144,145^, for an *i*-th node, the $$M\left[ m_{b} \right] $$ local measurement is set in bases $$m_{b} \in \left\{ m_{0} ,m_{1} \right\} $$, as

35 $$\begin{aligned} M\left[ m_{0} \right] ={\left| \psi _{0} \right\rangle } {\left\langle 0 \right| } , \end{aligned}$$

and

36 $$\begin{aligned} M\left[ m_{1} \right] ={\left| \psi _{1} \right\rangle } {\left\langle 1 \right| } , \end{aligned}$$

where $${\left| \psi _{0} \right\rangle } =\cos {\textstyle \frac{\varsigma }{2}} {\left| 0 \right\rangle } +e^{i\alpha } \sin {\textstyle \frac{\varsigma }{2}} {\left| 1 \right\rangle } $$, $${\left| \psi _{1} \right\rangle } =\sin {\textstyle \frac{\varsigma }{2}} {\left| 0 \right\rangle } -e^{i\alpha } \cos {\textstyle \frac{\varsigma }{2}} {\left| 1 \right\rangle } $$, with $$\varsigma \in \left[ 0,\pi \right] $$. (Assuming that the entangled connections between the nodes are maximally entangled, $$\varsigma =\pi $$, and $$\varsigma <\pi $$ otherwise. This parameter is also referred to as entanglement factor, see also^76^). Then, it can be verified^76,143^ that by applying $$M\left[ m_{b} \right] $$ local measurements in the $$L-2$$ intermediate nodes between $$A_{i} $$ and $$B_{i} $$ as defined by (35) and (36), Bob $$B_{i} $$ obtains the result $$U({\vec{\theta}}_{i}){\left| + \right\rangle } _{i} $$ with probability $$\Pr \left( U({\vec{\theta}}_{i}){\left| + \right\rangle } _{i} \right) =1-\cos {\textstyle \frac{\varsigma }{2}} $$.

Therefore, applying the measurement procedure in the intermediate nodes of the *n* computational paths, results in (28) at the receiver side in a distributed manner, as

37 $$\begin{aligned} {\left| \phi ^{*} \right\rangle } ={{\mathcal L}}_{D} \left( U\left( N\right) {\textstyle \frac{1}{\sqrt{2^{n} } }} \left( \left( {\left| 0 \right\rangle } \right) _{n+1}^{n2\left( L-1\right) } +\left( {\left| 1 \right\rangle } \right) _{n+1}^{n2\left( L-1\right) } \right) \right) , \end{aligned}$$

with probability

38 $$\begin{aligned} \Pr \left( {\left| \phi ^{*} \right\rangle } \right) =\prod _{i=1}^{n}\left( 1-\cos {\textstyle \frac{\varsigma _{i} }{2}} \right) \end{aligned}$$

over the *n* paths. Thus, if the network is maximally entangled it yields a deterministic download at the receiver with $\Pr \left( {\left| \phi ^{*} \right\rangle } \right) =1$.

The proof is concluded here. $\square$

**Figure 1:**
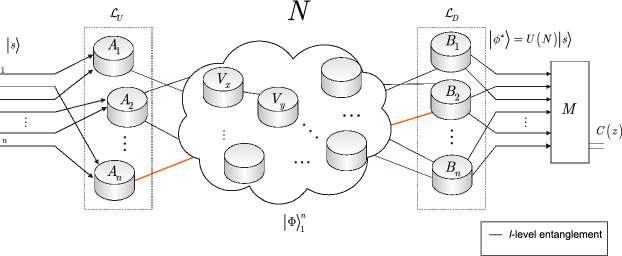
The schematic model of the distributed physical system *N* that realizes a scalable distributed quantum computation with arbitrary-level entangled connections. The $${\left| s \right\rangle } ={\left| + \right\rangle } _{1} \ldots {\left| + \right\rangle } _{n} $$ input system is distributed via *n* source nodes $$A_{1} ,\ldots ,A_{n} $$ through a chain of intermediate nodes via *l*-level entangled connections to the *n* receiver nodes $$B_{1} ,\ldots ,B_{n} $$, where $${\left| + \right\rangle } ={\textstyle \frac{1}{\sqrt{2} }} \left( {\left| 0 \right\rangle } +{\left| 1 \right\rangle } \right) $$, $${\left| s \right\rangle } ={\textstyle \frac{1}{\sqrt{2^{n} } }} \sum _{z}{\left| z \right\rangle } $$, while $${\left| z \right\rangle } $$ is an *n*-qubit length computational basis state. The aim of the distributed network system *N* is to maximize a *C* objective function of a computational problem in a distributed manner. The distributed system realizes the distributed unitary $$U\left( N\right) $$ and outputs a distributed system $${\left| \phi ^{*} \right\rangle } =U\left( N\right) {\left| s \right\rangle } $$. The *M* distributed measurements are performed in the *n* receiver nodes $$B_{1} ,\ldots ,B_{n} $$ to produce the string *z* that allows the nodes to evaluate $$C\left( z\right) $$ in a distributed way. The $${\left| s \right\rangle } $$ input system is uploaded via the $${{\mathcal L}}_{U} \left( {\left| s \right\rangle } \right) ={{\mathcal L}}_{U} \left( {\left| + \right\rangle } _{1} \ldots {\left| + \right\rangle } _{n} \right) $$ distributed uploading process to the distributed network state $${\left| \Phi \right\rangle } _{1}^{n} =U\left( N\right) {\textstyle \frac{1}{\sqrt{2} }} \left( \left( {\left| 00 \right\rangle } \right) _{1}^{n2\left( L-1\right) } +\left( {\left| 11 \right\rangle } \right) _{1}^{n2\left( L-1\right) } \right) =U\left( N\right) {\textstyle \frac{1}{\sqrt{2} }} \left( {\left| 0 \right\rangle } _{1}^{n} \left( {\left| 0 \right\rangle } \right) _{n+1}^{n2\left( L-1\right) } +{\left| 1 \right\rangle } _{1}^{n} \left( {\left| 1 \right\rangle } \right) _{n+1}^{n2\left( L-1\right) } \right) $$, where $${\left| \Phi \right\rangle } _{i} =U\left({\vec{\theta}}_{i} \right) {\textstyle \frac{1}{\sqrt{2} }} \left( {\left| 0 \right\rangle } _{1} \left( {\left| 0 \right\rangle } \right) _{2}^{2\left( L-1\right) } +{\left| 1 \right\rangle } _{1} \left( {\left| 1 \right\rangle } \right) _{2}^{2\left( L-1\right) } \right) $$, where index 1 identifies the first particle of computational path $${\mathcal {P}}\left( A_{i} \rightarrow B_{i} \right) $$, formulated via the results of the unitaries of the *n* computational paths, where an *i*-th path $${\mathcal {P}}\left( A_{i} \rightarrow B_{i} \right) $$, $$i=1,\ldots ,n$$, realizes an $$U\left({\vec{\theta}}_{i} \right) =U_{i,L} U_{i,L-1} \ldots U_{i,1} $$ unitary sequence of 2*L* unitaries in *L* nodes $$V_{x} $$, $$x=1,\ldots ,L$$, where $$U_{i,1} =U\left( \beta _{i,1} ,X_{i,1} \right) U\left( \gamma _{i,1} ,Z_{i,1} \right) $$. The $${{\mathcal L}}_{U} \left( {\left| s \right\rangle } \right) ={{\mathcal L}}_{U} \left( {\left| + \right\rangle } _{1} \right) \ldots {{\mathcal L}}_{U} \left( {\left| + \right\rangle } _{n} \right) $$ uploading process is distributed among the *n* nodes, where $${{\mathcal L}}_{U} \left( {\left| + \right\rangle } _{i} \right) $$ is realized in an *i*-th source node $$A_{i} $$ as $${{\mathcal L}}_{U} \left( \alpha _{i} {\left| 0 \right\rangle } +\beta _{i} {\left| 1 \right\rangle } \right) =U\left({\vec{\theta}}_{i} \right) \left( \alpha _{i} \left( {\left| 0 \right\rangle } \right) _{2}^{2\left( L-1\right) } +\beta _{i} \left( {\left| 1 \right\rangle } \right) _{2}^{2\left( L-1\right) } \right) =U\left({\vec{\theta}}_{i} \right) {\textstyle \frac{1}{\sqrt{2} }} \left( \left( {\left| 0 \right\rangle } \right) _{2}^{2\left( L-1\right) } +\left( {\left| 1 \right\rangle } \right) _{2}^{2\left( L-1\right) } \right) $$. The $${{\mathcal L}}_{D} $$ downloading process results in $${{\mathcal L}}_{D} \left( U\left({\vec{\theta}}_{i} \right) {\textstyle \frac{1}{\sqrt{2} }} \left( \left( {\left| 0 \right\rangle } \right) _{2}^{2\left( L-1\right) } +\left( {\left| 1 \right\rangle } \right) _{2}^{2\left( L-1\right) } \right) \right) =U\left({\vec{\theta}}_{i} \right) {\left| + \right\rangle } _{i} $$ for receiver node $$B_{i} $$. Applying $${{\mathcal L}}_{U} $$ and $${{\mathcal L}}_{D} $$ for all source and receiver nodes, results in $${{\mathcal L}}_{D} \left( {{\mathcal L}}_{U} \left( {\left| + \right\rangle } _{1} \ldots {\left| + \right\rangle } _{n} \right) \right) =U\left( N\right) {\left| s \right\rangle } $$ at the receiver in a distributed manner.

The system model of the scalable distributed physical system *N* is depicted in Fig. 1.

**Figure 2:**
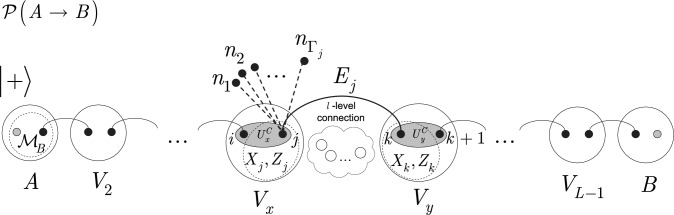
Evaluation of target function value *F* of a computational problem via a distributed computational path $${\mathcal {P}}\left( A\rightarrow B\right) $$ between a distant source node *A* and receiver node *B* in a distributed system *N* with $$L-2$$ intermediate nodes $$V_{x} $$, $$x=2,\ldots ,L-1$$, with multipartite entanglement in the local nodes. Alice applies an $${{\mathcal M}}_{B} $$ Bell measurement on the input system $${\left| + \right\rangle } $$ and on the first particle of the chain to achieve the $${{\mathcal L}}_{U} \left( {\left| + \right\rangle } \right) $$ uploading procedure. A node pair $$V_{xy} =\left\{ V_{x} ,V_{y} \right\} $$ with a shared *l*-level entangled connection $$E_{j} $$, $$j=1,\ldots ,L-1$$ ($$l=1$$ for a small-scale system while $$l\ge 1$$ for a medium- and large-scale system by a convention) is allowed to (1) apply a local coupling unitary $$U_{x}^{C} =\exp \left( -itH^{\left( i,j\right) } \right) $$ and $$U_{y}^{C} =\exp \left( -itH^{\left( k,k+1\right) } \right) $$ to connect qubits *i* (connected to $$V_{x-1} $$) to and *j* in $$V_{x} $$, and qubits *k* and $$k+1$$ (connected to $$V_{x+1} $$), (2) to perform a local single-qubit unitaries $$U\left( X_{j} ,\beta _{j} \right) $$ and $$U\left( X_{k} ,\beta _{k} \right) $$ on qubits *j* and *k* in $$V_{x} $$ and $$V_{y} $$, (3) to realize a distributed two-qubit unitary $$U\left( Z_{j} Z_{k} ,\gamma _{jk} \right) $$ on qubits *j* and *k* using the *l*-level entangled connection $$E_{j} $$, and (4) to apply an $$M\left[ m_{b} \right] $$ in basis $$m_{b} \in \left\{ m_{0} ,m_{1} \right\} $$, local measurement to realize the $${{\mathcal L}}_{D} $$ download into *B*. In a given $$V_{x} $$, qubit *j* formulates a multipartite entanglement: *j* has an entangled connection with qubit *k* in $$V_{y} $$ , and *j* is also entangled with $$\Gamma _{j} $$ other neighbor qubits, $$n_{1} ,\ldots ,n_{\Gamma _{j} } $$, called remote entangled connections of *j* (not neighbors of qubit *k*), and the total number of qubits that are neighbors of *j* but not neighbors of *k* is $$\Gamma _{j} +1$$. Each entangled connection $$E_{j} $$ has a contribution $$\zeta _{E_{j} } $$ to the expected target function value $$F_{{\mathcal {P}}\left( A\rightarrow B\right) } ={\textstyle \frac{1}{2}} \sum _{j=1}^{L-1}\zeta _{E_{j} } $$. (Operations associated with a particular qubit in a given node are depicted by dashed circles.)

The schematic model of a computational path ${\mathcal {P}}\left( A\rightarrow B\right)$ in a distributed physical system *N* is depicted in Fig. 2. (Local measurements of downloading procedure are not shown.)

### Ethics statement

This work did not involve any active collection of human data.

## Quantum processing in a distributed quantum system

### **Theorem 1**

*An objective function* *C* *can be maximized in the distributed quantum system* *N* *via a target function* $F=\sum _{\left\langle jk\right\rangle \in N}F_{\langle jk \rangle } =\mathop {\max }\limits _{\forall \theta } {\left\langle \phi ^{*} \left| C\right| \phi ^{*} \right\rangle }$, *where* $\left\langle jk\right\rangle$ *is an* *l*-*level*, $l\ge 1$, *entangled connection between qubits* *j* *and* *k*.

### *Proof*

Let *N* be the physical distributed quantum system, with a particular objective function *C* of a computational problem subject of a maximization. To simplify the discussion in the following section, allow us to focus on a single computational path ${\mathcal {P}}\left( A\rightarrow B\right)$, thus we set $n=1$, and $N={\mathcal {P}}\left( A\rightarrow B\right)$ with ${\left| s \right\rangle } ={\left| + \right\rangle }$; however, the derivations and results are not restricted to this case.

Let $U(\vec {\theta })$ be the unitary realized via the computational path ${\mathcal {P}}\left( A\rightarrow B\right)$, as

39 $$\begin{aligned} U\left({\vec{\theta}}\right) =U_{2L} \left( \theta _{2L} \right) U_{2L-1} \left( \theta _{2L-1} \right) \cdots U_{1} \left( \theta _{1} \right) , \end{aligned}$$

where $$i=1,\ldots ,2L$$, *L* is the number of nodes of *N* (number of distributed subsystems), 2*L* is the total number of unitaries in the *L* nodes (each node is defined via 2 unitaries) $$\theta _{i} $$ is a gate parameter associated with $$U_{i} $$, i.e., $$\theta _{i} =\beta _{i} $$ or $$\theta _{i} =\gamma _{i} $$, and $$\vec {\theta }$$ is the gate parameter vector defined as

40 $$\begin{aligned}{\vec{\theta}}=\left( \theta _{1} ,\ldots ,\theta _{2L-1} ,\theta _{2L} \right) ^{T} . \end{aligned}$$

Assuming that *N* consist of *g* single-qubit unitaries and *m* two-qubit unitaries for the entangled qubit pairs (*m* qubit pair connection in *N*), such that

41 $$\begin{aligned} L=g+m, \end{aligned}$$

unitary $U(\vec {\theta })$ from (39) can be rewritten as

42 $$\begin{aligned} U\left({\vec{\theta}}\right) =U\left( B,\vec {\beta }\right) U\left( C,\vec {\gamma }\right) , \end{aligned}$$

where

43 $$\begin{aligned} U\left( B,\vec {\beta }\right) =\prod _{j=1}^{g}U\left( B_{j} ,\beta _{j} \right) , \end{aligned}$$

where $\vec {\beta }$ is the gate parameter vector,

44 $$\begin{aligned} \vec {\beta }=\left( \beta _{1} ,\ldots ,\beta _{g} \right) ^{T} , \end{aligned}$$

and

45 $$\begin{aligned} B=\sum _{j}B_{j} \end{aligned}$$

where $$B_{j} =X_{j} =\sigma _{x}^{j} $$^12,14^, and

46 $$\begin{aligned} U\left( B_{j} ,\beta _{j} \right) =\exp \left( -i\beta _{j} X_{j} \right) , \end{aligned}$$

and $U\left( C,\vec {\gamma }\right)$ is defined as^14^

47 $$\begin{aligned} U\left( C,\vec {\gamma }\right) =\prod _{\left\langle jk\right\rangle \in N}U\left( C_{jk} ,\gamma _{jk} \right) , \end{aligned}$$

where $\left\langle jk\right\rangle \in N$ is an *l*-level, $l\ge 1$, entangled connection between qubits *j* and *k*, with gate parameter vector

$$\begin{aligned} \vec {\gamma }=\left( \gamma _{1} ,\ldots ,\gamma _{m} \right) ^{T} , \end{aligned}$$

and

48 $$\begin{aligned} U\left( C_{jk} ,\gamma _{jk} \right) =U\left( Z_{j} Z_{k} ,\gamma _{jk} C_{jk} \right) =\exp \left( -i\gamma _{jk} C_{jk} Z_{j} Z_{k} \right) , \end{aligned}$$

where $$Z_{j} Z_{k} =\sigma _{z}^{j} \sigma _{z}^{k} $$.

At a particular physical entangled connection topology in *N*, the objective function *C* can be written as

49 $$\begin{aligned} C\left( z\right) =\sum _{\left\langle jk\right\rangle \in N}C_{jk} \left( z\right) , \end{aligned}$$

where $C_{jk} \left( z\right)$ is the objective function component^12,14^ evaluated for entangled connection $\left\langle jk\right\rangle \in N$, as

50 $$\begin{aligned} C_{jk} \left( z\right) ={\textstyle \frac{1}{2}} \left( 1-z_{j} z_{k} \right) , \end{aligned}$$

where *z* is an *n*-length input bitstring,

51 $$\begin{aligned} z=z_{1} z_{2} \ldots z_{n} , \end{aligned}$$

and $$z_{i} $$ identifies an *i*-th bit, $$z_{i} \in \left\{ -1,1\right\} $$.

For a given *z*, a ${\left| z \right\rangle }$ computational basis state is defined as

52 $$\begin{aligned} {\left| z \right\rangle } ={\left| z_{1} z_{2} \ldots z_{n} \right\rangle } \end{aligned}$$

and ${\left| \varphi \right\rangle }$ output system of *N* at a single path at input (52) is defined as (For a level-*p* circuit, a set of *p* $\vec {\beta }$ and $\vec {\gamma }$ gate parameter vectors are used as ${{\vec {\beta }}^{\left( 1 \right) }},\ldots ,{{\vec {\beta }}^{\left( p \right) }}$, and ${{\vec {\gamma }}^{\left( 1 \right) }},\ldots ,{{\vec {\gamma }}^{\left( p \right) }}$. For simplicity, here we assume $p=1$, however the results can be extended for arbitrary *p*^14^. For further details, see^14^.)

53 $$\begin{aligned} \begin{aligned} \left| \varphi \right\rangle&=U\left( {\vec {\theta }}\right) \left| z \right\rangle \\&=U\left( B,\vec {\beta }\right) U\left( C,\vec {\gamma } \right) \left| z \right\rangle \\&=U\left( B,\vec {\beta }\right) \prod \limits _{\left\langle jk \right\rangle \in N}{U\left( {{C}_{jk}}\left( z \right) ,{{\gamma }_{jk}}\right) }\left| z \right\rangle \\&=\prod \limits _{j}{\exp \left( -i{{\beta }_{j}}{{X}_{j}} \right) }\prod \limits _{\left\langle jk \right\rangle \in N}{\exp \left( -i{{\gamma }_{jk}}{{C}_{jk}}\left( z \right) {{Z}_{j}}{{Z}_{k}} \right) }\left| z \right\rangle . \end{aligned} \end{aligned}$$

Then, let ${\left| s \right\rangle }$ be an *n*-qubit length input system of *N*, defined as in (19), thus for $n=1$,

54 $$\begin{aligned} {\left| s \right\rangle } ={\left| + \right\rangle } , \end{aligned}$$

and the output system ${\left| \varphi ^{*} \right\rangle }$ is evaluated as given in (17).

The maximization of objective function *C* is identified via a target function *F*, as

55 $$\begin{aligned} \begin{aligned} F&=\underset{\forall \theta }{\mathop {\max }}\,\left\langle \vec {\gamma },\vec {\beta },\left. C \right| \left. C \right| \vec {\gamma },\vec {\beta },C \right\rangle \\&=\underset{\forall \theta }{\mathop {\max }}\,\left\langle {{\varphi }^{*}} | \left. C \right| {{\varphi }^{*}} \right\rangle , \end{aligned} \end{aligned}$$

and for a particular entangled connection $\left\langle jk\right\rangle$ of *N*, the aim is the maximization of target function $F_{\langle jk \rangle }$, as

56 $$\begin{aligned} F_{\langle jk \rangle }=\mathop {\max }\limits _{\forall \theta } \left( \left( -{\textstyle \frac{1}{2}} \right) {\left\langle \varphi _{N,jk}^{*} \left| Z_{j} Z_{k} \right| \varphi _{N,jk}^{*} \right\rangle } \right) , \end{aligned}$$

where ${\left| \varphi _{N,jk}^{*} \right\rangle }$ is a target state defined as

57 $$\begin{aligned} \begin{aligned} \left| \varphi _{N,jk}^{*} \right\rangle&=\left| {{\gamma }_{jk}},{{\beta }_{k}},{{\beta }_{j}},{{C}_{jk}} \right\rangle \\&=U\left( B,{{\beta }_{j}} \right) U\left( B,{{\beta }_{k}} \right) U\left( {{C}_{jk}}\left( z \right) ,{{\gamma }_{jk}} \right) \left| s \right\rangle . \end{aligned} \end{aligned}$$

For the total system *N*, the objective function values of all entangled connections are summed, thus $C\left( z\right)$ is as given in (49).

For all connected qubits, the target function is set as

58 $$\begin{aligned} \begin{aligned} F&=\sum \limits _{\left\langle jk \right\rangle \in N}{F_{\langle jk \rangle }} \\&=\underset{\forall \theta }{\mathop {\max }}\,\left\langle {{\varphi }^{*}} | \left. C \right| {{\varphi }^{*}} \right\rangle \\&=\underset{\forall \left\langle jk \right\rangle \in N}{\mathop {\max }}\,\left( \left( -\tfrac{1}{2} \right) \sum \limits _{\left\langle jk \right\rangle \in N}{\left\langle \varphi _{N,jk}^{*} | \left. {{C}_{jk}} \right| \varphi _{N,jk}^{*} \right\rangle } \right) \\&=\left( -\tfrac{1}{2} \right) \underset{\forall \left\langle jk \right\rangle \in N}{\mathop {\max }}\,\left( \sum \limits _{\left\langle jk \right\rangle \in N}{\left\langle \varphi _{N,jk}^{*} | \left. {{Z}_{j}}{{Z}_{k}} \right| \varphi _{N,jk}^{*} \right\rangle } \right) , \end{aligned} \end{aligned}$$

where ${\left| \varphi _{N,jk}^{*} \right\rangle }$ is given in (57).

Then, assuming that *N* consists of *n* computational paths, and ${\left| s \right\rangle }$ is an *n*-qubit length input as defined in (19), the result in (58) can be extended as

59 $$\begin{aligned} \begin{aligned} F&=\sum \limits _{\left\langle jk \right\rangle \in N}{F_{\langle jk \rangle }} \\&=\underset{\forall \theta }{\mathop {\max }}\,\left\langle {{\phi }^{*}} | \left. C \right| {{\phi }^{*}} \right\rangle , \end{aligned} \end{aligned}$$

that concludes the proof. $\square$

### Distributed computational system as an extended correlation space

#### **Lemma 1**

(The distributed computational space is an extended correlation space). *The* ${{\mathcal D}}\left( N\right)$ *distributed computational space of* *N* *is an extended correlation space with* *n* *entangled computational paths*, ${\mathcal {P}}\left( A_{i} \rightarrow B_{i} \right)$, $i=1,\ldots ,n$ *between n source and receiver nodes*.

#### *Proof*

Using the correlation space (The correlation space is an abstract mathematical model of a physical system defined via a matrix product state (MPS) representation and open-boundary conditions^76,144,145^.) formalism^144,145^, we first rewrite ${\left| \varphi ^{*} \right\rangle }$ from (17), as

60 $$\begin{aligned} {\left| \varphi ^{*} \right\rangle } =\sum _{x_{1} ,\ldots ,x_{L} }{\left\langle B \right| } M\left[ x_{L} \right] M\left[ x_{L-1} \right] \ldots M\left[ x\right] {\left| A \right\rangle } {\left| x_{1} ,\ldots ,x_{L} \right\rangle } , \end{aligned}$$

where $$x_{i} \in \left\{ 0,1\right\} $$, $$i=1,\ldots ,L$$, $$M\left[ x_{i} \right] $$ is a $$2\times 2$$ matrix, $${\left| x_{i} \right\rangle } $$ is a local state vector associated to node $$V_{i} $$, as

61 $$\begin{aligned} {\left| x_{i} \right\rangle } =c_{0}^{\left( i\right) } {\left| 0 \right\rangle } +c_{1}^{\left( i\right) } {\left| 1 \right\rangle } , \end{aligned}$$

and $M\left[ x_{i} \right]$ is defined as

62 $$\begin{aligned} M\left[ x_{i} \right] =\bar{c}_{0}^{\left( i\right) } M\left[ 0\right] +\bar{c}_{1}^{\left( i\right) } M\left[ 1\right] , \end{aligned}$$

with relation^76,144,145^

63 $$\begin{aligned} \left( {\left\langle x_{1} \right| } \otimes \ldots \otimes {\left\langle x_{L} \right| } \right) {\left| \psi _{L} \right\rangle } ={\left\langle x_{L} \right| } M\left[ x_{L-1} \right] \ldots M\left[ x_{1} \right] {\left| + \right\rangle } , \end{aligned}$$

where ${\left| + \right\rangle } ={\textstyle \frac{1}{\sqrt{2} }} \left( {\left| 0 \right\rangle } +{\left| 1 \right\rangle } \right)$, while ${\left| A \right\rangle }$ and ${\left| B \right\rangle }$ are $d=2$ dimensional vectors that represent the input and output systems (boundary conditions in the extended correlation space).

The system state of (60) can be rewritten as

64 $$\begin{aligned} {\left| \varphi ^{*} \right\rangle } =\sum _{x_{1} ,\ldots ,x_{L} }{\left\langle x_{L} \right| } M\left[ x_{L-1} \right] \ldots M\left[ x_{1} \right] {\left| + \right\rangle } {\left| x_{1} ,\ldots ,x_{L} \right\rangle } . \end{aligned}$$

By recalling Observation 2 from^145^, allows us to the define $\delta _{i}$ via the $\varsigma \in \left[ 0,\pi \right]$ measurement coefficient used in the definition of measurement operators (35) and (36), as

65 $$\begin{aligned} \delta _{i} =\arg \left( \sin \left( \omega _{i} \right) +\cos \left( \omega _{i} \right) \exp \left( i{\textstyle \frac{\varsigma }{2}} \right) \right) , \end{aligned}$$

where $\omega _{i}$ identifies computational bases ${\left| b_{\omega _{i} } \right\rangle } \in \left\{ {\left| 0_{\omega _{i} } \right\rangle } ,{\left| 1_{\omega _{i} } \right\rangle } \right\}$, as

66 $$\begin{aligned} \left| {{b}_{{{\omega }_{i}}}} \right\rangle =\left\{ \begin{matrix} \left| {{0}_{{{\omega }_{i}}}} \right\rangle =\sin \left( {{\omega }_{i}} \right) \left| 0 \right\rangle +\cos \left( {{\omega }_{i}} \right) \left| 1 \right\rangle \\ \left| {{1}_{{{\omega }_{i}}}} \right\rangle =\cos \left( {{\omega }_{i}} \right) \left| 0 \right\rangle -\sin \left( {{\omega }_{i}} \right) \left| 1 \right\rangle \\ \end{matrix} \right. . \end{aligned}$$

Using $\omega _{i}$ along with $\varsigma$, a diagonal matrix $D\left( \omega _{i} ,\varsigma \right)$ can be defined as

67 $$\begin{aligned} D\left( \omega _{i} ,\varsigma \right) =\sqrt{p_{i} } S\left( -2\delta _{i} \right) , \end{aligned}$$

where

68 $$\begin{aligned} S\left( x\right) =diag\left( e^{{\textstyle \frac{-ix}{2}} } ,e^{{\textstyle \frac{ix}{2}} } \right) , \end{aligned}$$

while $$p_{i} $$ is evaluated via (65) as

69 $$\begin{aligned} p_{i} =\left| \delta _{i} \right| ^{2} . \end{aligned}$$

Finally, by exploiting Observation 3 of^145^, leads to

70 $$\begin{aligned} U\left({\vec{\theta}}\right) =WS\left( \delta _{L} \right) WS\left( \delta _{L-1} \right) \ldots WS\left( \delta _{1} \right) , \end{aligned}$$

where *W* is a matrix set as

71 $$\begin{aligned} W=\exp \left( i\pi {\textstyle \frac{X}{\mho }} \right) , \end{aligned}$$

where $\mho$ is a coefficient, such that

72 $$\begin{aligned} WS\left( \delta _{j} \right) =U_{j} =U\left( X_{j} ,\beta _{j} \right) U\left( Z_{j} Z_{k} ,\gamma _{j} \right) , \end{aligned}$$

where $\delta _{j}$ is as given in (65) (with an index update).

Thus, the ${{\mathcal D}}\left( {\mathcal {P}}\left( A\rightarrow B\right) \right)$ computational path is the map of the physical computational path

73 $$\begin{aligned} {\mathcal {P}}\left( A\rightarrow B\right) =U_{L} U_{L-1} \ldots U_{1} \end{aligned}$$

formulated via the *L* nodes $V_{1} ,\ldots ,V_{L}$ of *N* onto the correlation space, as

74 $$\begin{aligned} {{\mathcal D}}\left( {\mathcal {P}}\left( A\rightarrow B\right) \right) =WS\left( \delta _{L} \right) WS\left( \delta _{L-1} \right) \ldots WS\left( \delta _{1} \right) . \end{aligned}$$

Then using the system characterization of *N* of Section 2, reveals that ${{\mathcal D}}\left( N\right)$ is an extended correlation space with *n* computational paths, where an *i*-th computational path is evaluated as (74), thus the ${{\mathcal D}}\left( N\right)$ computational model of *N* is evaluated as

75 $$\begin{aligned} \begin{aligned} \mathcal {D}\left( N \right) =&\left( {{W}_{1}}S\left( {{\delta }_{1,L}} \right) {{W}_{1}}S\left( {{\delta }_{1,L-1}} \right) \ldots {{W}_{1}}S\left( {{\delta }_{1,1}} \right) \right) \ldots \\&\ldots \left( {{W}_{n}}S\left( {{\delta }_{n,L}} \right) {{W}_{n}}S\left( {{\delta }_{n,L-1}} \right) \ldots {{W}_{n}}S\left( {{\delta }_{n,1}} \right) \right) , \end{aligned} \end{aligned}$$

where $\left( i,j\right)$ identifies a *j*-th unitary of an *i*-th computational path ${\mathcal {P}}\left( A_{i} \rightarrow B_{i} \right)$, that concludes the proof. $\square$

### Objective function evaluation at multipartite entanglement

#### **Proposition 4**

*Let* $F_{{\mathcal {P}}\left( A\rightarrow B\right) }$ *be the target function at a given objective function* $C_{{\mathcal {P}}\left( A\rightarrow B\right) }$ *evaluated for the computational path* ${\mathcal {P}}\left( A\rightarrow B\right)$ *via* (58), *as*

76 $$\begin{aligned} \begin{aligned} {{F}_{\mathcal {P}\left( A\rightarrow B \right) }}&=\sum \limits _{\left\langle jk \right\rangle \in \mathcal {P}\left( A\rightarrow B \right) }{F_{\langle jk \rangle }} \\&=\underset{\forall \theta }{\mathop {\max }}\,\left\langle {{\varphi }^{*}} | \left. {{C}_{\mathcal {P}\left( A\rightarrow B \right) }} \right| {{\varphi }^{*}} \right\rangle \\&=\left( -\tfrac{1}{2} \right) \underset{\forall \left\langle jk \right\rangle \in \mathcal {P}\left( A\rightarrow B \right) }{\mathop {\max }}\,\left( \sum \limits _{\left\langle jk \right\rangle \in \mathcal {P}\left( A\rightarrow B \right) }{\left\langle \varphi _{N,jk}^{*} | \left. {{Z}_{j}}{{Z}_{k}} \right| \varphi _{N,jk}^{*} \right\rangle } \right) , \end{aligned} \end{aligned}$$

*where* $C_{{\mathcal {P}}\left( A\rightarrow B\right) }$ *is defined as*

77 $$\begin{aligned} C_{{\mathcal {P}}\left( A\rightarrow B\right) } =\sum _{\left\langle jk\right\rangle \in {\mathcal {P}}\left( A\rightarrow B\right) }C_{jk}. \end{aligned}$$

#### **Theorem 2**

(Scaling via gate parameters of unitaries). *The* $F_{{\mathcal {P}}\left( A\rightarrow B\right) }$ *target function of a computational path* ${\mathcal {P}}\left( A\rightarrow B\right)$ *with objective function* $C_{{\mathcal {P}}\left( A\rightarrow B\right) } =\sum _{\left\langle jk\right\rangle \in {\mathcal {P}}\left( A\rightarrow B\right) }C_{jk}$ *is maximized at gate parameters* $\beta _{j} ={\textstyle \frac{\pi }{8}}$ *and* $\gamma _{jk} ={\textstyle \frac{1}{2}} \cos ^{-1} \left( {\textstyle \frac{\Gamma _{j} -1}{\Gamma _{j} +1}} \right)$ *in the* *L* *nodes, where* $\Gamma _{j}$ *is the number of remote entangled connections of* *j*.

#### *Proof*

The proof utilizes the system model of Section 2, and focuses on a particular computational path ${\mathcal {P}}\left( A\rightarrow B\right)$ with an $C_{{\mathcal {P}}\left( A\rightarrow B\right) }$ objective function of a computational problem.

At $L-1$ entangled connections, $F_{{\mathcal {P}}\left( A\rightarrow B\right) }$ from (76) can be written as

78 $$\begin{aligned} F_{{\mathcal {P}}\left( A\rightarrow B\right) } ={\textstyle \frac{1}{2}} \sum _{j=1}^{L-1}\zeta _{E_{j} } , \end{aligned}$$

where $$\zeta _{E_{j} } $$ is the contribution of an *l*-level $$E_{j} $$ entangled connection between qubits *j* and *k* in target function $$F_{{\mathcal {P}}\left( A\rightarrow B\right) } $$, defined as

79 $$\begin{aligned} \zeta _{E_{j} } =\left( \sin \left( 2\beta _{j} +2\beta _{k} \right) \right) \sin \gamma _{jk} \prod _{k=1}^{\Gamma _{j} +1}\cos \gamma _{jk} , \end{aligned}$$

where $$\Gamma _{j} $$ is the number of remote neighbor entangled qubits of *j* such that not neighbors of qubit *k*, while $$\beta _{j} $$, $$\beta _{k} $$ and $$\gamma _{jk} $$ are the gate parameters of unitaries of $$U_{xy} $$ in (13) (The evaluation of (79) utilizes an abstraction. The structure of the distributed system is mapped onto a grid such that the vertices of the grid represent the qubits in the nodes, while an edge between the qubits identifies an *l*-level $$E_{j} $$ entangled connection in the distributed system. Since all connections between the qubits are entangled, the vertices on the grid are separated only by the particular edge that directly connects the qubits, thus the distance between the qubits on the grid is set to unit for all connections^14^.)

Assuming that $\gamma _{jk}$ is set to the same value for all *k*, $k=1,\ldots ,\Gamma _{j} +1$ , at $\beta _{j} =\beta _{k}$ the result in (79) can be simplified as

80 $$\begin{aligned} \zeta _{E_{j} } =\left( \sin 4\beta _{j} \right) \sin \gamma _{jk} \cos ^{\left( \Gamma _{j} +1\right) } \gamma _{jk} . \end{aligned}$$

To verify (79), we first rewrite (56) for a particular entangled connection $$E_{j} $$, as

81 $$\begin{aligned} \begin{aligned} F_{\langle jk \rangle }&=\left( -\tfrac{1}{2} \right) \left\langle \varphi _{N,jk}^{*} | \left. {{Z}_{j}}{{Z}_{k}} \right| \varphi _{N,jk}^{*} \right\rangle \\&=\left( -\tfrac{1}{2} \right) \left\langle s \right| {{U}^{\dagger }}\left( N,{{\gamma }_{jk}} \right) \left( {{U}^{\dagger }}\left( {{\beta }_{j}},{{X}_{j}} \right) {{Z}_{j}}U\left( {{\beta }_{j}},{{X}_{j}} \right) \right) \left( {{U}^{\dagger }}\left( {{\beta }_{k}},{{X}_{k}} \right) {{Z}_{k}}U\left( {{\beta }_{k}},{{X}_{k}} \right) \right) U\left( N,{{\gamma }_{jk}} \right) \left| + \right\rangle , \end{aligned} \end{aligned}$$

where ${\left| \varphi _{N,jk}^{*} \right\rangle }$ is the target state from (57), and

82 $$\begin{aligned} U\left( \beta _{j} ,X_{j} \right) =\exp \left( -i\beta _{j} X_{j} \right) =\cos \left( \beta _{j} \right) I-i\sin \left( \beta _{j} \right) X_{j} \end{aligned}$$

and

83 $$\begin{aligned} U\left( N,\gamma _{jk} \right) =\exp \left( -i\gamma _{jk} Z_{j} Z_{k} \right) =\cos \left( \gamma _{jk} \right) I-i\sin \left( \gamma _{jk} \right) Z_{j} Z_{k} , \end{aligned}$$

where *N* is a product of pairs of *Z* operators^12,14^.

Then,

84 $$\begin{aligned} \begin{aligned}    & {{U}^{\dagger }}\left( {{\beta }_{j}},{{X}_{j}} \right) {{Z}_{j}}U\left( {{\beta }_{j}},{{X}_{j}} \right) \left( {{U}^{\dagger }}\left( {{\beta }_{k}},{{X}_{k}} \right) {{Z}_{k}}U\left( {{\beta }_{k}},{{X}_{k}} \right) \right) \\&\quad =\exp \left( i{{\beta }_{j}}{{X}_{j}} \right) {{Z}_{j}}\exp \left( -i{{\beta }_{j}}{{X}_{j}} \right) \exp \left( i{{\beta }_{k}}{{X}_{k}} \right) {{Z}_{k}}\exp \left( -i{{\beta }_{k}}{{X}_{k}} \right) , \end{aligned} \end{aligned}$$

where

85 $$\begin{aligned} \begin{aligned}    & \exp \left( i{{\beta }_{j}}{{X}_{j}} \right) {{Z}_{j}}\exp \left( -i{{\beta }_{j}}{{X}_{j}} \right) \\&\quad =\left( \cos \left( {{\beta }_{j}} \right) I+i\sin \left( {{\beta }_{j}} \right) {{X}_{j}} \right) {{Z}_{j}}\left( \cos \left( {{\beta }_{j}} \right) I-i\sin \left( {{\beta }_{j}} \right) {{X}_{j}} \right) \\&\quad =\left( \cos \left( {{\beta }_{j}} \right) {{Z}_{j}}+i\sin \left( {{\beta }_{j}} \right) {{Z}_{j}}{{X}_{j}} \right) \left( \cos \left( {{\beta }_{j}} \right) I-i\sin \left( {{\beta }_{j}} \right) {{X}_{j}} \right) \\&\quad =\left( \cos \left( {{\beta }_{j}} \right) {{Z}_{j}}+i\sin \left( {{\beta }_{j}} \right) {{Y}_{j}} \right) \left( \cos \left( {{\beta }_{j}} \right) I-i\sin \left( {{\beta }_{j}} \right) {{X}_{j}} \right) \\&\quad ={{\cos }^{2}}\left( {{\beta }_{j}} \right) {{Z}_{j}}-i\cos \left( {{\beta }_{j}} \right) \sin \left( {{\beta }_{j}} \right) {{Z}_{j}}{{X}_{j}}+i\cos \left( {{\beta }_{j}} \right) \sin \left( {{\beta }_{j}} \right) {{Y}_{j}}-{{i}^{2}}{{\sin }^{2}}\left( {{\beta }_{j}} \right) {{Y}_{j}}{{X}_{j}} \\&\quad ={{\cos }^{2}}\left( {{\beta }_{j}} \right) {{Z}_{j}}+i\cos \left( {{\beta }_{j}} \right) \sin \left( {{\beta }_{j}} \right) {{X}_{j}}{{Z}_{j}}+i\cos \left( {{\beta }_{j}} \right) \sin \left( {{\beta }_{j}} \right) {{Y}_{j}}-{{i}^{2}}{{\sin }^{2}}\left( {{\beta }_{j}} \right) {{Y}_{j}}{{X}_{j}} \\&\quad ={{\cos }^{2}}\left( {{\beta }_{j}} \right) {{Z}_{j}}+i\cos \left( {{\beta }_{j}} \right) \sin \left( {{\beta }_{j}} \right) {{Y}_{j}}+i\cos \left( {{\beta }_{j}} \right) \sin \left( {{\beta }_{j}} \right) {{Y}_{j}}+{{i}^{2}}{{\sin }^{2}}\left( {{\beta }_{j}} \right) {{Z}_{j}} \\&\quad =\left( {{\cos }^{2}}\left( {{\beta }_{j}} \right) {{Z}_{j}}+{{i}^{2}}{{\sin }^{2}}\left( {{\beta }_{j}} \right) {{Z}_{j}} \right) +2i\cos \left( {{\beta }_{j}} \right) \sin \left( {{\beta }_{j}} \right) {{Y}_{j}} \\&\quad =\left( \tfrac{1}{2}\left( 1+\cos \left( 2{{\beta }_{j}} \right) \right) {{Z}_{j}}-\tfrac{1}{2}\left( 1-\cos \left( 2{{\beta }_{j}} \right) \right) {{Z}_{j}} \right) +2i\left( \tfrac{1}{2}\left( \sin \left( 2{{\beta }_{j}} \right) -\sin \left( 0 \right) \right) \right) {{Y}_{j}} \\&\quad =\left( \tfrac{1}{2}{{Z}_{j}}+\tfrac{1}{2}\cos \left( 2{{\beta }_{j}} \right) {{Z}_{j}}-\tfrac{1}{2}{{Z}_{j}}+\tfrac{1}{2}\cos \left( 2{{\beta }_{j}} \right) {{Z}_{j}} \right) +i\sin \left( 2{{\beta }_{j}} \right) {{Y}_{j}} \\&\quad ={{Z}_{j}}\cos \left( 2{{\beta }_{j}} \right) +i{{Y}_{j}}\sin \left( 2{{\beta }_{j}} \right) , \end{aligned} \end{aligned}$$

and

86 $$\begin{aligned} \begin{aligned}    & \exp \left( i{{\beta }_{k}}{{X}_{k}} \right) {{Z}_{k}}\exp \left( -i{{\beta }_{k}}{{X}_{k}} \right) \\&\quad =\left( \cos \left( {{\beta }_{k}} \right) I+i\sin \left( {{\beta }_{k}} \right) {{X}_{k}} \right) {{Z}_{k}}\left( \cos \left( {{\beta }_{k}} \right) I-i\sin \left( {{\beta }_{k}} \right) {{X}_{k}} \right) \\&\quad =\left( \cos \left( {{\beta }_{k}} \right) {{Z}_{j}}+i\sin \left( {{\beta }_{k}} \right) {{Z}_{k}}{{X}_{k}} \right) \left( \cos \left( {{\beta }_{k}} \right) I-i\sin \left( {{\beta }_{k}} \right) {{X}_{k}} \right) \\&\quad =\left( \cos \left( {{\beta }_{k}} \right) {{Z}_{k}}+i\sin \left( {{\beta }_{k}} \right) {{Y}_{k}} \right) \left( \cos \left( {{\beta }_{k}} \right) I-i\sin \left( {{\beta }_{k}} \right) {{X}_{k}} \right) \\&\quad ={{\cos }^{2}}\left( {{\beta }_{k}} \right) {{Z}_{k}}-i\cos \left( {{\beta }_{k}} \right) \sin \left( {{\beta }_{k}} \right) {{Z}_{j}}{{X}_{j}}+i\cos \left( {{\beta }_{k}} \right) \sin \left( {{\beta }_{k}} \right) {{Y}_{k}}-{{i}^{2}}{{\sin }^{2}}\left( {{\beta }_{k}} \right) {{Y}_{k}}{{X}_{k}} \\&\quad ={{\cos }^{2}}\left( {{\beta }_{k}} \right) {{Z}_{k}}+i\cos \left( {{\beta }_{k}} \right) \sin \left( {{\beta }_{k}} \right) {{X}_{j}}{{Z}_{j}}+i\cos \left( {{\beta }_{k}} \right) \sin \left( {{\beta }_{k}} \right) {{Y}_{k}}-{{i}^{2}}{{\sin }^{2}}\left( {{\beta }_{k}} \right) {{Y}_{k}}{{X}_{k}} \\&\quad ={{\cos }^{2}}\left( {{\beta }_{k}} \right) {{Z}_{k}}+i\cos \left( {{\beta }_{k}} \right) \sin \left( {{\beta }_{k}} \right) {{Y}_{k}}+i\cos \left( {{\beta }_{k}} \right) \sin \left( {{\beta }_{k}} \right) {{Y}_{k}}+{{i}^{2}}{{\sin }^{2}}\left( {{\beta }_{k}} \right) {{Z}_{k}} \\&\quad =\left( {{\cos }^{2}}\left( {{\beta }_{k}} \right) {{Z}_{k}}+{{i}^{2}}{{\sin }^{2}}\left( {{\beta }_{k}} \right) {{Z}_{k}} \right) +2i\cos \left( {{\beta }_{k}} \right) \sin \left( \beta \right) {{Y}_{k}} \\&\quad =\left( \tfrac{1}{2}\left( 1+\cos \left( 2{{\beta }_{k}} \right) \right) {{Z}_{k}}-\tfrac{1}{2}\left( 1-\cos \left( 2{{\beta }_{k}} \right) \right) {{Z}_{k}} \right) +2i\left( \tfrac{1}{2}\left( \sin \left( 2{{\beta }_{k}} \right) -\sin \left( 0 \right) \right) \right) {{Y}_{k}} \\&\quad =\left( \tfrac{1}{2}{{Z}_{k}}+\tfrac{1}{2}\cos \left( 2{{\beta }_{k}} \right) {{Z}_{k}}-\tfrac{1}{2}{{Z}_{k}}+\tfrac{1}{2}\cos \left( 2{{\beta }_{k}} \right) {{Z}_{k}} \right) +i\sin \left( 2{{\beta }_{k}} \right) {{Y}_{k}} \\&\quad ={{Z}_{k}}\cos \left( 2{{\beta }_{k}} \right) +i{{Y}_{k}}\sin \left( 2{{\beta }_{k}} \right) , \end{aligned} \end{aligned}$$

thus

87 $$\begin{aligned} \begin{aligned}    & {{U}^{\dagger }}\left( {{\beta }_{j}},{{X}_{j}} \right) {{Z}_{j}}U\left( {{\beta }_{j}},{{X}_{j}} \right) {{U}^{\dagger }}\left( {{\beta }_{k}},{{X}_{k}} \right) {{Z}_{k}}U\left( {{\beta }_{k}},{{X}_{k}} \right) \\&\quad =\left( {{Z}_{j}}\cos \left( 2{{\beta }_{j}} \right) +i{{Y}_{j}}\sin \left( 2{{\beta }_{j}} \right) \right) \left( {{Z}_{k}}\cos \left( 2{{\beta }_{k}} \right) +i{{Y}_{k}}\sin \left( 2{{\beta }_{k}} \right) \right) . \end{aligned} \end{aligned}$$

Assuming, that $\beta _{j} =\beta _{k} =\beta$, (87) can be written in a simplified form as

88 $$\begin{aligned} \begin{aligned}    & U{{\left( \beta ,X \right) }^{\dagger }}{{Z}_{j}}{{Z}_{k}}U\left( \beta ,X \right) \\&\quad ={{U}^{\dagger }}\left( {{\beta }_{j}},{{X}_{j}} \right) {{Z}_{j}}U\left( {{\beta }_{j}},{{X}_{j}} \right) {{U}^{\dagger }}\left( {{\beta }_{k}},{{X}_{k}} \right) {{Z}_{k}}U\left( {{\beta }_{k}},{{X}_{k}} \right) \\&\quad ={{\cos }^{2}}\left( 2\beta \right) {{Z}_{j}}{{Z}_{k}}+i\cos \left( 2\beta \right) \sin \left( 2\beta \right) {{Z}_{j}}{{Y}_{k}}+i\cos \left( 2\beta \right) \sin \left( 2\beta \right) {{Y}_{j}}{{Z}_{k}}+{{i}^{2}}{{\sin }^{2}}\left( 2\beta \right) {{Y}_{j}}{{Y}_{k}}. \end{aligned} \end{aligned}$$

The related terms $U\left( N,\gamma _{jk} \right) Z_{j} U^{\dag } \left( N,\gamma _{jk} \right)$ and $U\left( N,\gamma _{jk} \right) Y_{j} U^{\dag } \left( N,\gamma _{jk} \right)$ of (81) are evaluated as

89 $$\begin{aligned} \begin{aligned}    & U\left( N,{{\gamma }_{jk}} \right) {{Z}_{j}}{{U}^{\dagger }}\left( N,{{\gamma }_{jk}} \right) \\&\quad =\left( \cos \left( {{\gamma }_{jk}} \right) I-i\sin \left( {{\gamma }_{jk}} \right) {{Z}_{j}}{{Z}_{k}} \right) {{Z}_{j}}\left( \cos \left( {{\gamma }_{jk}} \right) I+i\sin \left( {{\gamma }_{jk}} \right) {{Z}_{j}}{{Z}_{k}} \right) \\&\quad =\left( {{Z}_{j}}\cos \left( {{\gamma }_{jk}} \right) I-i{{Z}_{j}}\sin \left( {{\gamma }_{jk}} \right) {{Z}_{j}}{{Z}_{k}} \right) \left( \cos \left( {{\gamma }_{jk}} \right) I+i\sin \left( {{\gamma }_{jk}} \right) {{Z}_{j}}{{Z}_{k}} \right) \\&\quad =\left( \cos \left( {{\gamma }_{jk}} \right) {{Z}_{j}}-i\sin \left( {{\gamma }_{jk}} \right) {{Z}_{k}} \right) \left( \cos \left( {{\gamma }_{jk}} \right) I+i\sin \left( {{\gamma }_{jk}} \right) {{Z}_{j}}{{Z}_{k}} \right) \\&\quad ={{\cos }^{2}}\left( {{\gamma }_{jk}} \right) {{Z}_{j}}+i\cos \left( {{\gamma }_{jk}} \right) \sin \left( {{\gamma }_{jk}} \right) {{Z}_{k}}-i\cos \left( {{\gamma }_{jk}} \right) \sin \left( {{\gamma }_{jk}} \right) {{Z}_{k}}-{{i}^{2}}{{\sin }^{2}}\left( {{\gamma }_{jk}} \right) {{Z}_{j}} \\&\quad ={{\cos }^{2}}\left( {{\gamma }_{jk}} \right) {{Z}_{j}}-{{i}^{2}}{{\sin }^{2}}\left( {{\gamma }_{jk}} \right) {{Z}_{j}} \\&\quad ={{\cos }^{2}}\left( {{\gamma }_{jk}} \right) {{Z}_{j}}+{{\sin }^{2}}\left( {{\gamma }_{jk}} \right) {{Z}_{j}} \\&\quad =\tfrac{1}{2}\left( 1+\cos \left( 2{{\gamma }_{jk}} \right) \right) {{Z}_{j}}+\tfrac{1}{2}\left( 1-\cos \left( 2{{\gamma }_{jk}} \right) \right) {{Z}_{j}} \\&\quad =\tfrac{1}{2}{{Z}_{j}}+\cos \left( 2{{\gamma }_{jk}} \right) {{Z}_{j}}+\tfrac{1}{2}{{Z}_{j}}-\cos \left( 2{{\gamma }_{jk}} \right) {{Z}_{j}} \\&={{Z}_{j}}, \end{aligned} \end{aligned}$$

and

90 $$\begin{aligned} \begin{aligned}    & U\left( N,{{\gamma }_{jk}} \right) {{Y}_{j}}{{U}^{\dagger }}\left( 
N,{{\gamma }_{jk}} \right) \\&\quad =\left( 
\cos \left( {{\gamma }_{jk}} \right) I-i\sin \left( {{\gamma }_{jk}} \right) {{Z}_{j}}{{Z}_{k}} \right) {{Y}_{j}}\left( \cos \left( {{\gamma }_{jk}} \right) I+i\sin \left( {{\gamma }_{jk}} \right) {{Z}_{j}}{{Z}_{k}} \right) \\&\quad =\left( \cos \left( {{\gamma }_{jk}} \right) {{Y}_{j}}-i\sin \left( {{\gamma }_{jk}} \right) {{Y}_{j}}{{Z}_{j}}{{Z}_{k}} \right) \left( \cos \left( {{\gamma }_{jk}} \right) I+i\sin \left( {{\gamma }_{jk}} \right) {{Z}_{j}}{{Z}_{k}} \right) \\&\quad =\left( \cos \left( {{\gamma }_{jk}} \right) {{Y}_{j}}-i\sin \left( {{\gamma }_{jk}} \right) {{X}_{j}}{{Z}_{k}} \right) \left( \cos \left( {{\gamma }_{jk}} \right) I+i\sin \left( {{\gamma }_{jk}} \right) {{Z}_{j}}{{Z}_{k}} \right) \\&\quad ={{\cos }^{2}}\left( {{\gamma }_{jk}} \right) {{Y}_{j}}+i\cos \left( {{\gamma }_{jk}} \right) \sin \left( {{\gamma }_{jk}} \right) {{Y}_{j}}{{Z}_{j}}{{Z}_{k}}-i\sin \left( {{\gamma }_{jk}} \right) \cos \left( {{\gamma }_{jk}} \right) {{X}_{j}}{{Z}_{k}}-{{i}^{2}}{{\sin }^{2}}\left( {{\gamma }_{jk}} \right) \left( {{X}_{j}}{{Z}_{k}}{{Z}_{j}}{{Z}_{k}} \right) \\&\quad ={{\cos }^{2}}\left( {{\gamma }_{jk}} \right) {{Y}_{j}}-i\cos \left( {{\gamma }_{jk}} \right) \sin \left( {{\gamma }_{jk}} \right) \left( {{X}_{j}}{{Z}_{j}} \right) {{Z}_{j}}{{Z}_{k}}-i\sin \left( {{\gamma }_{jk}} \right) \cos \left( {{\gamma }_{jk}} \right) {{X}_{j}}{{Z}_{k}}+{{i}^{2}}{{\sin }^{2}}\left( {{\gamma }_{jk}} \right) \left( {{Y}_{j}} \right) \\&\quad ={{\cos }^{2}}\left( {{\gamma }_{jk}} \right) {{Y}_{j}}-i\cos \left( {{\gamma }_{jk}} \right) \sin \left( {{\gamma }_{jk}} \right) {{X}_{j}}{{Z}_{k}}-i\sin \left( {{\gamma }_{jk}} \right) \cos \left( {{\gamma }_{jk}} \right) {{X}_{j}}{{Z}_{k}}+{{i}^{2}}{{\sin }^{2}}\left( {{\gamma }_{jk}} \right) \left( {{Y}_{j}} \right) \\&\quad =\left( \tfrac{1}{2}\left( 1+\cos \left( 2{{\gamma }_{jk}} \right) \right) {{Y}_{j}}-\tfrac{1}{2}\left( 1-\cos \left( 2{{\gamma }_{jk}} \right) \right) {{Y}_{j}} \right) -2i\left( \tfrac{1}{2}\left( \sin \left( 2{{\gamma }_{jk}} \right) -\sin \left( 0 \right) \right) \right) {{X}_{j}}{{Z}_{k}} \\&\quad =\left( \tfrac{1}{2}{{Y}_{j}}+\tfrac{1}{2}\cos \left( 2{{\gamma }_{jk}} \right) {{Y}_{j}}-\tfrac{1}{2}{{Y}_{j}}+\tfrac{1}{2}\cos \left( 2{{\gamma }_{jk}} \right) {{Y}_{j}} \right) -2i\left( \tfrac{1}{2}\left( \sin \left( 2{{\gamma }_{jk}} \right) -\sin \left( 0 \right) \right) \right) {{X}_{j}}{{Z}_{k}} \\&\quad ={{Y}_{j}}\cos \left( 2{{\gamma }_{jk}} \right) -i{{X}_{j}}{{Z}_{k}}\sin \left( 2{{\gamma }_{jk}} \right) . \end{aligned} \end{aligned}$$

Then, using (81), let $\chi _{jk}$ be defined as

91 $$\begin{aligned} \chi _{jk} =U^{\dag } \left( N,\gamma _{jk} \right) \left( U^{\dag } \left( \beta _{j} ,X_{j} \right) Z_{j} U\left( \beta _{j} ,X_{j} \right) \right) \left( U^{\dag } \left( \beta _{k} ,X_{k} \right) Z_{k} U\left( \beta _{k} ,X_{k} \right) \right) U\left( N,\gamma _{jk} \right) \end{aligned}$$

thus (81) can be rewritten as

92 $$\begin{aligned} F_{\langle jk \rangle }=\left( -{\textstyle \frac{1}{2}} \right) {\left\langle + \right| } \chi _{jk} {\left| + \right\rangle } , \end{aligned}$$

with

93 $$\begin{aligned} {\left\langle + \right| } X{\left| + \right\rangle } =1, \end{aligned}$$

and

94 $$\begin{aligned} {\left\langle + \right| } Z{\left| + \right\rangle } ={\left\langle + \right| } Y{\left| + \right\rangle } =0. \end{aligned}$$

It can be verified^14^, that $\chi _{jk}$ can be decomposed as

95 $$\begin{aligned} \chi _{jk} =\eta _{j} \eta _{k} , \end{aligned}$$

where

96 $$\begin{aligned} \begin{aligned} {{\eta }_{j}}&={{Z}_{j}}\cos 2{{\beta }_{j}}+\left( {{Y}_{j}}\cos {{\gamma }_{jk}}-{{X}_{j}}{{Z}_{k}}\sin {{\gamma }_{jk}} \right) \sin 2{{\beta }_{j}}\prod \limits _{k=1}^{{{\Gamma }_{j}}+1}{\cos {{\gamma }_{jk}}} \\&={{Z}_{j}}\cos 2{{\beta }_{j}}+\left( {{Y}_{j}}\sin 2{{\beta }_{j}}\prod \limits _{k=1}^{{{\Gamma }_{j}}}{\cos {{\gamma }_{jk}}}-{{X}_{j}}{{Z}_{k}}\sin {{\gamma }_{jk}}\sin 2{{\beta }_{j}}\prod \limits _{k=1}^{{{\Gamma }_{j}}+1}{\cos {{\gamma }_{jk}}} \right) \end{aligned} \end{aligned}$$

and

97 $$\begin{aligned} \begin{aligned} {{\eta }_{k}}&={{Z}_{k}}\cos 2{{\beta }_{k}}+\left( {{Y}_{k}}\cos {{\gamma }_{jk}}-{{X}_{k}}{{Z}_{j}}\sin {{\gamma }_{jk}} \right) \sin 2{{\beta }_{k}}\prod \limits _{k=1}^{{{\Gamma }_{j}}+1}{\cos {{\gamma }_{jk}}} \\&={{Z}_{k}}\cos 2{{\beta }_{k}}+\left( {{Y}_{k}}\sin 2{{\beta }_{k}}\prod \limits _{k=1}^{{{\Gamma }_{j}}}{\cos {{\gamma }_{jk}}}-{{X}_{k}}{{Z}_{j}}\sin {{\gamma }_{jk}}\sin 2{{\beta }_{k}}\prod \limits _{k=1}^{{{\Gamma }_{j}}+1}{\cos {{\gamma }_{jk}}} \right) . \end{aligned} \end{aligned}$$

Thus, $\chi _{jk}$ can be evaluated as

98 $$\begin{aligned} \begin{aligned} {{\chi }_{jk}}=&\left( {{Z}_{j}}\cos 2{{\beta }_{j}}+{{Y}_{j}}\sin 2{{\beta }_{j}}\prod \limits _{k=1}^{{{\Gamma }_{j}}}{\cos {{\gamma }_{jk}}}-{{X}_{j}}{{Z}_{k}}\sin {{\gamma }_{jk}}\sin 2{{\beta }_{j}}\prod \limits _{k=1}^{{{\Gamma }_{j}}+1}{\cos {{\gamma }_{jk}}} \right) \\&\times \left( {{Z}_{k}}\cos 2{{\beta }_{k}}+{{Y}_{k}}\sin 2{{\beta }_{k}}\prod \limits _{k=1}^{{{\Gamma }_{j}}}{\cos {{\gamma }_{jk}}}-{{X}_{k}}{{Z}_{j}}\sin {{\gamma }_{jk}}\sin 2{{\beta }_{k}}\prod \limits _{k=1}^{{{\Gamma }_{j}}+1}{\cos {{\gamma }_{jk}}} \right) \\ =&{{Z}_{j}}{{Z}_{k}}\cos 2{{\beta }_{j}}\cos 2{{\beta }_{k}}+{{Z}_{j}}{{Y}_{k}}\cos 2{{\beta }_{j}}\sin 2{{\beta }_{k}}\prod \limits _{k=1}^{{{\Gamma }_{j}}}{\cos {{\gamma }_{jk}}} \\&-{{X}_{k}}\cos 2{{\beta }_{j}}\sin {{\gamma }_{jk}}\sin 2{{\beta }_{k}}\prod \limits _{k=1}^{{{\Gamma }_{j}}+1}{\cos {{\gamma }_{jk}}} \\&+{{Y}_{j}}{{Z}_{k}}\sin 2{{\beta }_{j}}\prod \limits _{k=1}^{{{\Gamma }_{j}}}{\cos {{\gamma }_{jk}}}\cos 2{{\beta }_{k}}+{{Y}_{j}}{{Y}_{k}}\sin 2{{\beta }_{j}}\sin 2{{\beta }_{k}}\prod \limits _{k=1}^{2{{\Gamma }_{j}}}{\cos {{\gamma }_{jk}}} \\&-{{Y}_{j}}{{Z}_{j}}{{X}_{k}}\sin {{\gamma }_{jk}}\sin 2{{\beta }_{j}}\sin 2{{\beta }_{k}}\prod \limits _{k=1}^{2{{\Gamma }_{j}}+1}{\cos {{\gamma }_{jk}}} \\&-{{X}_{j}}\sin {{\gamma }_{jk}}\sin 2{{\beta }_{j}}\cos 2{{\beta }_{k}}\prod \limits _{k=1}^{{{\Gamma }_{j}}+1}{\cos {{\gamma }_{jk}}}-{{X}_{j}}{{Z}_{k}}{{Y}_{k}}\sin {{\gamma }_{jk}}\sin 2{{\beta }_{j}}\sin 2{{\beta }_{k}}\prod \limits _{k=1}^{2{{\Gamma }_{j}}+1}{\cos {{\gamma }_{jk}}} \\&+{{X}_{j}}{{Z}_{j}}{{Z}_{k}}{{X}_{k}}{{\sin }^{2}}{{\gamma }_{jk}}\sin 2{{\beta }_{j}}\sin 2{{\beta }_{k}}\prod \limits _{k=1}^{2\left( {{\Gamma }_{j}}+1 \right) }{\cos {{\gamma }_{jk}}} \\ =&{{Z}_{j}}{{Z}_{k}}\cos 2{{\beta }_{j}}\cos 2{{\beta }_{k}}+{{Z}_{j}}{{Y}_{k}}\cos 2{{\beta }_{j}}\sin 2{{\beta }_{k}}\prod \limits _{k=1}^{{{\Gamma }_{j}}}{\cos {{\gamma }_{jk}}} \\&-{{X}_{k}}\cos 2{{\beta }_{j}}\sin {{\gamma }_{jk}}\sin 2{{\beta }_{k}}\prod \limits _{k=1}^{{{\Gamma }_{j}}+1}{\cos {{\gamma }_{jk}}}+{{Y}_{j}}{{Z}_{k}}\sin 2{{\beta }_{j}}\prod \limits _{k=1}^{{{\Gamma }_{j}}}{\cos {{\gamma }_{jk}}}\cos 2{{\beta }_{k}} \\&+{{Y}_{j}}{{Y}_{k}}\sin 2{{\beta }_{j}}\sin 2{{\beta }_{k}}\prod \limits _{k=1}^{2{{\Gamma }_{j}}}{\cos {{\gamma }_{jk}}}-{{X}_{j}}{{X}_{k}}\sin {{\gamma }_{jk}}\sin 2{{\beta }_{j}}\sin 2{{\beta }_{k}}\prod \limits _{k=1}^{2{{\Gamma }_{j}}+1}{\cos {{\gamma }_{jk}}} \\&-{{X}_{j}}\sin {{\gamma }_{jk}}\sin 2{{\beta }_{j}}\cos 2{{\beta }_{k}}\prod \limits _{k=1}^{{{\Gamma }_{j}}+1}{\cos {{\gamma }_{jk}}}+{{X}_{j}}{{X}_{k}}\sin {{\gamma }_{jk}}\sin 2{{\beta }_{j}}\sin 2{{\beta }_{k}}\prod \limits _{k=1}^{2{{\Gamma }_{j}}+1}{\cos {{\gamma }_{jk}}} \\&-{{Y}_{j}}{{Y}_{k}}{{\sin }^{2}}{{\gamma }_{jk}}\sin 2{{\beta }_{j}}\sin 2{{\beta }_{k}}\prod \limits _{k=1}^{2\left( {{\Gamma }_{j}}+1 \right) }{\cos {{\gamma }_{jk}}}. \end{aligned} \end{aligned}$$

Then, by utilizing the fact that input system ${\left| + \right\rangle }$, and therefore also ${\left| s \right\rangle }$, is an eigenstate of each *X* with eigenvalue 1^14^ (see also (93) and (94)), the terms containing *Y* and *Z* vanish from (98), while *X* can be replaced as $X=1$. As follows, (98) can be rewritten as

99 $$\begin{aligned} \begin{aligned} {{\chi 
}_{jk}}=&-{{X}_{k}}\cos 2{{\beta }_{j}}\sin {{\gamma }_{jk}}\sin 2{{\beta }_{k}}\prod \limits _{k=1}^{{{\Gamma }_{j}}+1}{\cos {{\gamma }_{jk}}} \\&-{{X}_{j}}{{X}_{k}}\sin {{\gamma }_{jk}}\sin 2{{\beta }_{j}}\sin 2{{\beta }_{k}}\prod \limits _{k=1}^{2{{\Gamma }_{j}}+1}{\cos {{\gamma }_{jk}}} \\&-{{X}_{j}}\cos 2{{\beta }_{k}}\sin {{\gamma }_{jk}}\sin 2{{\beta }_{j}}\prod \limits _{k=1}^{{{\Gamma }_{j}}+1}{\cos {{\gamma }_{jk}}}+{{X}_{j}}{{X}_{k}}\sin {{\gamma }_{jk}}\sin 2{{\beta }_{j}}\sin 2{{\beta }_{k}}\prod \limits _{k=1}^{2{{\Gamma }_{j}}+1}{\cos {{\gamma }_{jk}}} \\ =&-\left( {{X}_{k}}\cos 2{{\beta }_{j}}\sin {{\gamma }_{jk}}\sin 2{{\beta }_{k}}\prod \limits _{k=1}^{{{\Gamma }_{j}}+1}{\cos {{\gamma }_{jk}}}+{{X}_{j}}\cos 2{{\beta }_{k}}\sin {{\gamma }_{jk}}\sin 2{{\beta }_{j}}\prod \limits _{k=1}^{{{\Gamma }_{j}}+1}{\cos {{\gamma }_{jk}}} \right) \\ =&-\left( \cos 2{{\beta }_{j}}\sin {{\gamma }_{jk}}\sin 2{{\beta }_{k}}\prod \limits _{k=1}^{{{\Gamma }_{j}}+1}{\cos {{\gamma }_{jk}}}+\cos 2{{\beta }_{k}}\sin {{\gamma }_{jk}}\sin 2{{\beta }_{j}}\prod \limits _{k=1}^{{{\Gamma }_{j}}+1}{\cos {{\gamma }_{jk}}} \right) . \end{aligned} \end{aligned}$$

Further assuming that $\beta _{j} =\beta _{k} =\beta$ holds, (99) can be simplified as

100 $$\begin{aligned} \chi _{jk} =-2\cos 2\beta \sin \gamma _{jk} \sin 2\beta \prod _{k=1}^{\Gamma _{j} +1}\cos \gamma _{jk} . \end{aligned}$$

Therefore, (92) is as

101 $$\begin{aligned} \begin{aligned}    & F_{\langle jk \rangle } \\&\quad =-\tfrac{1}{2}{{\chi }_{jk}} \\&\quad =\tfrac{1}{2}\left( \cos 2{{\beta }_{j}}\sin 2{{\beta }_{k}}+\cos 2{{\beta }_{k}}\sin 2{{\beta }_{j}} \right) \left( \sin {{\gamma }_{jk}}\prod \limits _{k=1}^{{{\Gamma }_{j}}+1}{\cos {{\gamma }_{jk}}} \right) \\&\quad =\tfrac{1}{2}\left( \sin \left( 2{{\beta }_{j}}+2{{\beta }_{k}} \right) -\tfrac{1}{2}\sin \left( 2{{\beta }_{j}}-2{{\beta }_{k}} \right) -\tfrac{1}{2}\sin \left( 2{{\beta }_{k}}-2{{\beta }_{j}} \right) \right) \left( \sin {{\gamma }_{jk}}\prod \limits _{k=1}^{{{\Gamma }_{j}}+1}{\cos {{\gamma }_{jk}}} \right) \\&\quad =\tfrac{1}{2}\sin \left( 2{{\beta }_{j}}+2{{\beta }_{k}} \right) \left( \sin {{\gamma }_{jk}}\prod \limits _{k=1}^{{{\Gamma }_{j}}+1}{\cos {{\gamma }_{jk}}} \right) , \end{aligned} \end{aligned}$$

which at condition $\beta _{j} =\beta _{k} =\beta$ (which is the case for a maximization) simplifies as

102 $$\begin{aligned} \begin{aligned}    & F_{\langle jk \rangle }=\tfrac{1}{2}\sin \left( 4\beta \right) \left( \sin {{\gamma }_{jk}}\prod \limits _{k=1}^{{{\Gamma }_{j}}+1}{\cos {{\gamma }_{jk}}} \right) \\&\quad =\tfrac{1}{2}{{\zeta }_{{{E}_{j}}}}. \end{aligned} \end{aligned}$$

Then, using (79), the $C_{{\mathcal {P}}\left( A\rightarrow B\right) }$ objective function of the ${\mathcal {P}}\left( A\rightarrow B\right)$ computational path is evaluated as

103 $$\begin{aligned} C_{{\mathcal {P}}\left( A\rightarrow B\right) } ={\textstyle \frac{1}{2}} \phi _{{\mathcal {P}}\left( A\rightarrow B\right) } +{\textstyle \frac{1}{2}} \sum _{j=1}^{L-1}\zeta _{E_{j} } , \end{aligned}$$

where $\phi _{{\mathcal {P}}\left( A\rightarrow B\right) }$ identifies the total number of entangled connections of ${\mathcal {P}}\left( A\rightarrow B\right)$, as

104 $$\begin{aligned} \phi _{{\mathcal {P}}\left( A\rightarrow B\right) } ={\textstyle \frac{1}{2}} \left( \sum _{j=1}^{L-1}\left( \Gamma _{j} +2\right) \right) +{\textstyle \frac{1}{2}} , \end{aligned}$$

where the term $+{\textstyle \frac{1}{2}} ={\textstyle \frac{1}{2}} \left( +1\right)$ indicates the coupling unitary $U_{B}^{C} =\exp \left( -itH^{\left( k,B\right) } \right)$ in node *B* in the evaluation $C_{{\mathcal {P}}\left( A\rightarrow B\right) }$, by a convention.

Assuming that (80) holds, (103) is simplified as

105 $$\begin{aligned} \begin{aligned} {{C}_{\mathcal {P}\left( A\rightarrow B \right) }}&=\tfrac{1}{4}+\tfrac{1}{4}\sum \limits _{j=1}^{L-1}{\left( {{\Gamma }_{j}}+2 \right) }+\tfrac{1}{2}\sum \limits _{j=1}^{L-1}{\left( \sin 4{{\beta }_{j}} \right) \sin {{\gamma }_{jk}}{{\cos }^{\left( {{\Gamma }_{j}}+1 \right) }}{{\gamma }_{jk}}} \\&=\tfrac{1}{4}+\tfrac{1}{2}\sum \limits _{j=1}^{L-1}{\tfrac{1}{2}\left( {{\Gamma }_{j}}+2 \right) +\left( \sin 4{{\beta }_{j}} \right) \sin {{\gamma }_{jk}}{{\cos }^{\left( {{\Gamma }_{j}}+1 \right) }}{{\gamma }_{jk}}.} \end{aligned} \end{aligned}$$

If for each node the same $\beta _{j}$, $\gamma _{jk}$ and $\Gamma _{j}$ values are set, (105) can be rewritten as

106 $$\begin{aligned} C_{{\mathcal {P}}\left( A\rightarrow B\right) } ={\textstyle \frac{1}{4}} +{\textstyle \frac{1}{2}} \left( L-1\right) \left( {\textstyle \frac{1}{2}} \left( \Gamma _{j} +2\right) +\left( \sin 4\beta _{j} \right) \sin \gamma _{jk} \cos ^{\left( \Gamma _{j} +1\right) } \gamma _{jk} \right) . \end{aligned}$$

After some calculations, the gate-parameter values $\beta _{j}$ and $\gamma _{jk}$ that maximize $\zeta _{E_{j} }$ (and therefore $C_{{\mathcal {P}}\left( A\rightarrow B\right) }$) are at

107 $$\begin{aligned} 4\cos 4\beta _{j} =0 \end{aligned}$$

and

108 $$\begin{aligned} \cos ^{\left( \Gamma _{j} +2\right) } \gamma _{jk} -\left( \Gamma _{j} +1\right) \cos ^{\left( \Gamma _{j} \right) } \gamma _{jk} \sin ^{2} \gamma _{jk} =0, \end{aligned}$$

that yields gate parameter values

109 $$\begin{aligned} \beta _{j} ={\textstyle \frac{\pi }{8}} , \end{aligned}$$

and

110 $$\begin{aligned} \gamma _{jk} ={\textstyle \frac{1}{2}} \cos ^{-1} \left( {\textstyle \frac{\Gamma _{j} -1}{\Gamma _{j} +1}} \right) . \end{aligned}$$

Thus, (102) is maximized as

111 $$\begin{aligned} F_{\langle jk \rangle }={\textstyle \frac{1}{2}} \sin \left( {\textstyle \frac{\pi }{2}} \right) \left( \sin \left( {\textstyle \frac{1}{2}} \cos ^{-1} \left( {\textstyle \frac{\Gamma _{j} -1}{\Gamma _{j} +1}} \right) \right) \prod _{k=1}^{\Gamma _{j} +1}\cos \left( {\textstyle \frac{1}{2}} \cos ^{-1} \left( {\textstyle \frac{\Gamma _{j} -1}{\Gamma _{j} +1}} \right) \right) \right) . \end{aligned}$$

The maximized $C_{{\mathcal {P}}\left( A\rightarrow B\right) }$ objective function value of (105) for a given computational path is therefore

112 $$\begin{aligned} \begin{aligned} {{C}_{\mathcal {P}\left( A\rightarrow B \right) }}&=\tfrac{1}{4}+\tfrac{1}{2}\sum \limits _{j=1}^{L-1}{\tfrac{1}{2}\left( {{\Gamma }_{j}}+2 \right) +\left( \sin \tfrac{\pi }{2} \right) \left( \sin \left( \tfrac{1}{2}{{\cos }^{-1}}\left( \tfrac{{{\Gamma }_{j}}-1}{{{\Gamma }_{j}}+1} \right) \right) \right) {{\cos }^{\left( {{\Gamma }_{j}}+1 \right) }}\left( \tfrac{1}{2}{{\cos }^{-1}}\left( \tfrac{{{\Gamma }_{j}}-1}{{{\Gamma }_{j}}+1} \right) \right) } \\&=\tfrac{1}{4}+\tfrac{1}{2}\sum \limits _{j=1}^{L-1}{\tfrac{1}{2}\left( {{\Gamma }_{j}}+2 \right) +\sin \left( \tfrac{\pi }{4}-\tfrac{1}{2}{{\sin }^{-1}}\left( \tfrac{{{\Gamma }_{j}}-1}{{{\Gamma }_{j}}+1} \right) \right) {{\cos }^{\left( {{\Gamma }_{j}}+1 \right) }}\left( \tfrac{\pi }{4}-\tfrac{1}{2}{{\sin }^{-1}}\left( \tfrac{{{\Gamma }_{j}}-1}{{{\Gamma }_{j}}+1} \right) \right) ,} \end{aligned} \end{aligned}$$

and the maximized value of (106) is as

113 $$\begin{aligned} C_{{\mathcal {P}}\left( A\rightarrow B\right) } ={\textstyle \frac{1}{4}} +{\textstyle \frac{1}{2}} \left( L-1\right) \left( {\textstyle \frac{1}{2}} \left( \Gamma _{j} +2\right) +\sin \left( {\textstyle \frac{\pi }{4}} -{\textstyle \frac{1}{2}} \sin ^{-1} \left( {\textstyle \frac{\Gamma _{j} -1}{\Gamma _{j} +1}} \right) \right) \cos ^{\left( \Gamma _{j} +1\right) } \left( {\textstyle \frac{\pi }{4}} -{\textstyle \frac{1}{2}} \sin ^{-1} \left( {\textstyle \frac{\Gamma _{j} -1}{\Gamma _{j} +1}} \right) \right) \right) . \end{aligned}$$

The proof is concluded here. $\square$

**Figure 3:**
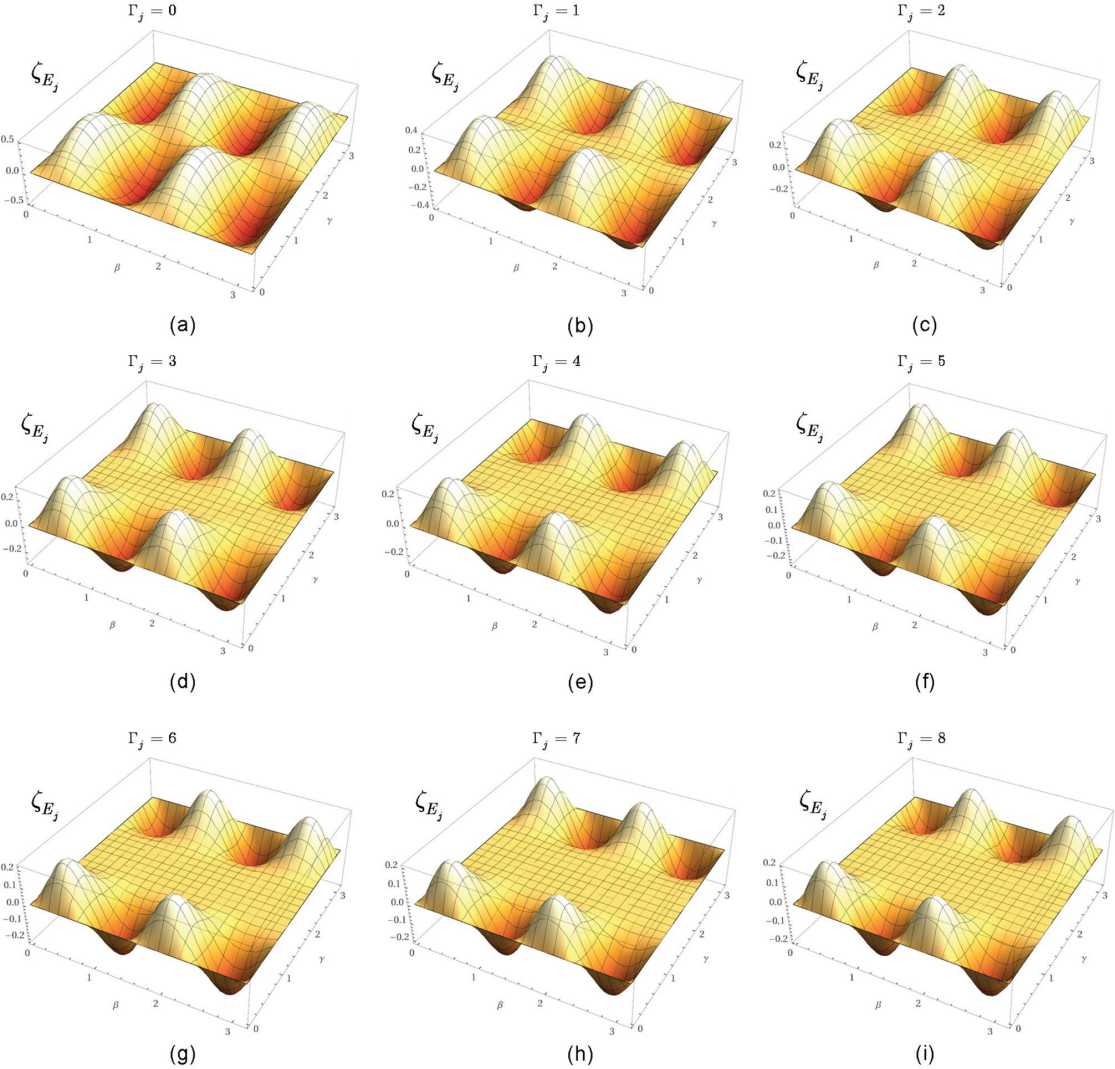
The values of $\zeta _{E_{j} }$ in function of gate parameters $\beta _{j}$ and $\gamma _{j} ={\textstyle \frac{1}{2}} \gamma _{jk}$, for different $\Gamma _{j}$ values ($\gamma _{jk}$ is set for the same value for all *k*, $k=1,\ldots ,\Gamma _{j} +1$). (**a**) $\Gamma _{j} =0$ (**b**) $\Gamma _{j} =1$ (**c**) $\Gamma _{j} =2$ (**d**) $\Gamma _{j} =3$ (**e**) $\Gamma _{j} =4$ (**f**) $\Gamma _{j} =5$ (**g**) $\Gamma _{j} =6$ (**h**) $\Gamma _{j} =7$ (**i**) $\Gamma _{j} =8$.

The values of $\zeta _{E_{j} }$ in function of gate parameters $\beta _{j}$ and $\gamma _{j} ={\textstyle \frac{1}{2}} \gamma _{jk}$, for different $\Gamma _{j}$ values ($\gamma _{jk}$ is set for the same value for all *k*, $k=1,\ldots ,\Gamma _{j} +1$) are depicted in Fig. 3.

**Figure 4:**
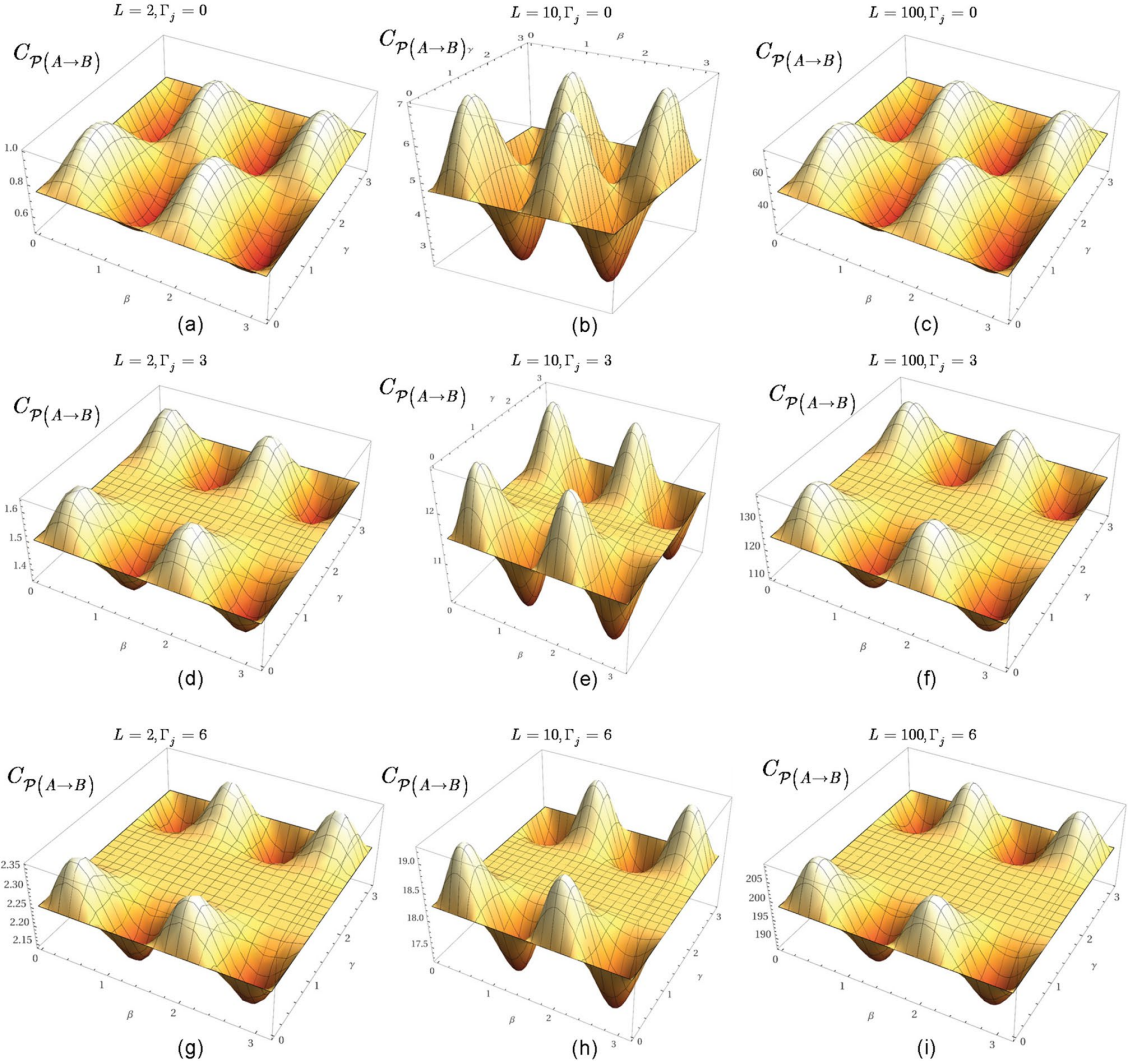
The $C_{{\mathcal {P}}\left( A\rightarrow B\right) }$ objective function values for a computational path ${\mathcal {P}}\left( A\rightarrow B\right)$ in function of gate parameters $\beta _{j}$ and $\gamma _{j} ={\textstyle \frac{1}{2}} \gamma _{jk}$, for different *L* and $\Gamma _{j}$ ($\beta _{j}$ and $\gamma _{j}$ are set for the same values for all *j*, $j=1,\ldots ,L-1$ and *k*, $k=1,\ldots ,\Gamma _{j} +1$). (**a**) $L=2,\Gamma _{j} =0$ (**b**) $L=10,\Gamma _{j} =0$ (**c**) $L=100,\Gamma _{j} =0$ (**d**) $L=2,\Gamma _{j} =3$ (**e**) $L=10,\Gamma _{j} =3$ (**f**) $L=100,\Gamma _{j} =3$ (**g**) $L=2,\Gamma _{j} =6$ (**h**) $L=10,\Gamma _{j} =6$ (**i**) $L=100,\Gamma _{j} =6$.

The objective function values (106) for a computational path ${\mathcal {P}}\left( A\rightarrow B\right)$ in function of gate parameters $\beta _{j}$ and $\gamma _{j} ={\textstyle \frac{1}{2}} \gamma _{jk}$, at different *L* node number and $\Gamma _{j}$ values ($\beta _{j}$ and $\gamma _{j}$ are set as the same for all *j*, $j=1,\ldots ,L-1$ and *k*, $k=1,\ldots ,\Gamma _{j} +1$) are depicted in Fig. 4.

**Figure 5:**
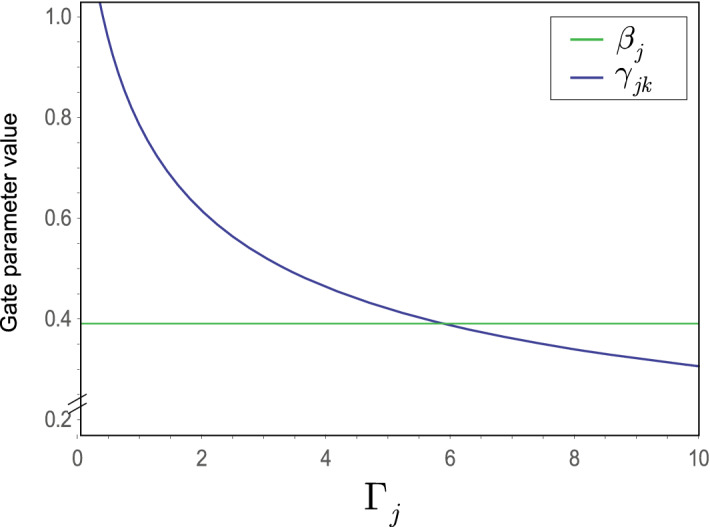
Gate parameter values $\beta _{j}$ and $\gamma _{jk}$ in function of $\Gamma _{j}$ for the maximization of $\zeta _{E_{j} }$ in the distributed system *N*.

The gate parameter values $\beta _{j}$ and $\gamma _{jk}$ for the maximization of $\zeta _{E_{j} }$ are depicted in Fig. 5.

## Scaling of a distributed quantum processing

### Target function scaling at a decoherence

Let ${{\mathcal S}}_{\varphi _{N,jk}^{*} } \left( t\right)$ be the time evolution^146^ of target state ${\left| \varphi _{N,jk}^{*} \left( t\right) \right\rangle }$ defined at a particular *t*, as

114 $$\begin{aligned} \begin{aligned} {{\mathcal {S}}_{\varphi _{N,jk}^{*}}}\left( t \right)&={{\left| \left\langle \left. \varphi _{N,jk}^{*}\left( {{t}_{0}} \right) \right| \varphi _{N,jk}^{*}\left( t \right) \right\rangle \right| }^{2}} \\&={{\left| \mathcal {A}\left( t \right) \right| }^{2}}, \end{aligned} \end{aligned}$$

where ${\left| \varphi _{N,jk}^{*} \left( t\right) \right\rangle }$ is defined via (57), as a target state at *t*, as

115 $$\begin{aligned} \begin{aligned} \left| \varphi _{N,jk}^{*}\left( t \right) \right\rangle&=\left| {{\gamma }_{jk}}\left( t \right) ,{{\beta }_{k}}\left( t \right) ,{{\beta }_{j}}\left( t \right) ,N \right\rangle \\&=U\left( B,{{\beta }_{j}}\left( t \right) \right) U\left( B,{{\beta }_{k}}\left( t \right) \right) U\left( N,{{\gamma }_{jk}}\left( t \right) \right) \left| s \right\rangle , \end{aligned} \end{aligned}$$

where $\beta _{j} \left( t\right)$, $\beta _{k} \left( t\right)$ and $\gamma _{jk} \left( t\right)$ are values of the gate parameters at a given time *t* associated to $\left\langle ij\right\rangle$, while ${\left| \varphi _{N,jk}^{*} \left( t_{0} \right) \right\rangle }$ is an initial state at some $t_{0} \in \left[ 0,T\right]$, while ${{\mathcal A}}\left( t\right)$ is the survival amplitude^146,147^, defined as

116 $$\begin{aligned} {{\mathcal A}}\left( t\right) =\left\langle \left. \varphi _{N,jk}^{*} \left( t_{0} \right) \right| \left. {\hat{U}}\left( t,t_{0} \right) \right| \varphi _{N,jk}^{*} \left( t_{0} \right) \right\rangle , \end{aligned}$$

where $\hat{U}\left( t,t_{0}\right)$ is the time evolution operator generated by a Hamiltonian $\hat{H}$, as

117 $$\begin{aligned} \hat{U}\left( t,t_{0} \right) =T\exp \left( -i\int \limits _{t_{0} }^{t}dx{\hat{H}\left( x\right) \bigg / \hbar } \right) . \end{aligned}$$

From the exponential decay law^147^, (116) can be written as

118 $$\begin{aligned} {{\mathcal A}}\left( t\right) =e^{-\Delta t} , \end{aligned}$$

where $\Delta$ is the decay rate^146^.

#### **Proposition 5**

*The* $F_{\left\langle jk\right\rangle } \left( t\right)$ *is the target function* $F_{\langle jk \rangle }$ *from* (56) *at a given* *t*, *defined as*

119 $$\begin{aligned} F_{\left\langle jk\right\rangle } \left( t\right) =\mathop {\max }\limits _{\forall \theta } \left( \left( -{\textstyle \frac{1}{2}} \right) {\left\langle \varphi _{N,jk}^{*} \left( t\right) \left| Z_{j} Z_{k} \right| \varphi _{N,jk}^{*} \left( t\right) \right\rangle } \right) . \end{aligned}$$

#### **Theorem 3**

(Target function scaling at decoherence). *At an systemal decoherence, for any non-zero quantum decay* $\Delta$ *on* $\left\langle ij\right\rangle$, *the* $F\left( \left\langle ij\right\rangle \right)$ *target function is scalable via the local* $M\left[ m_{b} \right]$ *measurement operator of the* ${{\mathcal L}}_{D}$ *download procedure*.

#### *Proof*

Let assume that the total number of entangled connections of *N* is $D=n\left( L-1\right)$. Then, let $t_{\left\langle ij\right\rangle } \left( N\right)$ be a vector of initialization time parameters of the target states of the entangled connections, defined as

120 $$\begin{aligned} t_{\left\langle ij\right\rangle } \left( N\right) =\left( t_{0}^{\left( 1\right) } ,\ldots ,t_{0}^{\left( D\right) } \right) ^{T} , \end{aligned}$$

where $t_{0}^{\left( j\right) } \in \left[ 0,T\right]$ is the initialization time (an initial time value when the target state is prepared) of target state ${\left| \varphi _{N,jk}^{*} \left( t_{0} \right) \right\rangle }$, $j=1,\ldots ,D$.

For the survival amplitudes of the system states associated to the entangled connections at a given *t*, we also define a ${{\mathcal A}}_{N} \left( t\right)$ vector of survival amplitudes associated to the *D* target states, as

121 $$\begin{aligned} {{\mathcal A}}_{N} \left( t\right) =\left( {{\mathcal A}}^{\left( 1\right) } \left( t\right) ,\ldots ,{{\mathcal A}}^{\left( D\right) } \left( t\right) \right) ^{T} =\left( e^{-\Delta _{1} t} ,\ldots ,e^{-\Delta _{D} t} \right) ^{T} , \end{aligned}$$

where ${{\mathcal A}}^{\left( j\right) } \left( t\right)$ is the survival amplitude of ${\left| \varphi _{N,jk}^{*} \left( t\right) \right\rangle }$, while $\Delta _{j}$ is the decay rate belongs to ${{\mathcal A}}^{\left( j\right) } \left( t\right)$ defined via $\hat{U}\left( t,t_{0}^{\left( j\right) } \right)$ that evolves ${\left| \varphi _{N,jk}^{*} \left( t_{0}^{\left( j\right) } \right) \right\rangle }$ to ${\left| \varphi _{N,jk}^{*} \left( t\right) \right\rangle }$.

Then, let ${{\mathcal L}}_{D}$ be a downloading procedure that requires the utilization of $M\left[ m_{b} \right]$ local measurements for the localization onto the target nodes with a measurement vector $M\left( N\right)$, as

122 $$\begin{aligned} M\left( N\right) =\left( M\left( \tau ^{\left( 1\right) } \right) \left[ m_{b} \right] ,\ldots ,M\left( \tau ^{\left( 2D\right) } \right) \left[ m_{b} \right] \right) ^{T} , \end{aligned}$$

where $M\left( \tau ^{\left( j\right) } \right) \left[ m_{b} \right]$ identifies a measurement $M\left[ m_{b} \right]$ on qubit *j* of $\left\langle jk\right\rangle$ at time $\tau ^{\left( i\right) } \in \left[ 0,T\right]$ in *N*, $i=1,\ldots ,2D$, as

123 $$\begin{aligned} M\left( {{\tau }^{\left( j \right) }} \right) \left[ {{m}_{b}} \right] =\left\{ \begin{matrix} 1,\text {if }j\text { is measured at }{{\tau }^{\left( j \right) }} \\ 0,\text {otherwise.} \\ \end{matrix} \right. \end{aligned}$$

Using (118), for a given qubit *j*, we define the $\mu _{j} \left( t\right)$ cumulated target state intensity which is dynamic term to model the interaction within the entangled network structure, as follows. Term $\mu _{j} \left( t\right)$ is defined as the sum of weighted target state decoherence terms (weighted target state intensities) of existing neighboring entangled connections and the actual weighted target state intensity at a local decoherence (local target function intensity), as

124 $$\begin{aligned} \begin{aligned} {{\mu }_{j}}\left( t \right)&={{\Lambda }_{\left\langle jk \right\rangle }}\left( t,t_{0}^{\left( j \right) } \right) +\sum \limits _{l=1,l\ne k}^{{{\Gamma }_{j}}+1}{{{\Lambda }_{\left\langle jl \right\rangle }}\left( t,t_{0}^{\left( l \right) }<t \right) } \\&={{F}_{\left\langle jk \right\rangle }}\left( t_{0}^{\left( j \right) } \right) {{\mathcal {A}}_{j}}\left( t,t_{0}^{\left( j \right) } \right) +\sum \limits _{l=1,l\ne k}^{{{\Gamma }_{j}}+1}{\int \limits _{0}^{t}{{{\Lambda }_{\left\langle jl \right\rangle }}\left( t,s \right) d{{G}_{l}}\left( s \right) }} \\&={{F}_{\left\langle jk \right\rangle }}\left( t_{0}^{\left( j \right) } \right) {{\mathcal {A}}_{j}}\left( t,t_{0}^{\left( j \right) } \right) +\sum \limits _{l=1,l\ne k}^{{{\Gamma }_{j}}+1}{\int \limits _{0}^{t}{{{F}_{\left\langle jl \right\rangle }}\left( t_{0}^{\left( l \right) } \right) {{\mathcal {A}}_{l}}\left( t,s \right) d{{G}_{l}}\left( s \right) }} \\&={{F}_{\left\langle jk \right\rangle }}\left( t_{0}^{\left( j \right) } \right) {{e}^{-{{\Delta }_{j}}\left( t-t_{0}^{\left( j \right) } \right) }}+\sum \limits _{l=1,l\ne k}^{{{\Gamma }_{j}}+1}{\int \limits _{0}^{t}{{{F}_{\left\langle jl \right\rangle }}\left( t_{0}^{\left( l \right) } \right) {{e}^{-{{\Delta }_{l}}\left( t-s \right) }}d{{G}_{l}}\left( s \right) ,}} \end{aligned} \end{aligned}$$

where term $\Lambda _{\left\langle jk\right\rangle } \left( t,t_{0}^{\left( j\right) } \right)$ is defined as the intensity of a target state ${\left| \varphi _{N,jk}^{*} \left( t\right) \right\rangle }$,

125 $$\begin{aligned} \begin{aligned} {{\Lambda }_{\left\langle jk \right\rangle }}\left( t,t_{0}^{\left( j \right) } \right)&={{F}_{\left\langle jk \right\rangle }}\left( t_{0}^{\left( j \right) } \right) {{\mathcal {A}}_{j}}\left( t,t_{0}^{\left( j \right) } \right) \\&={{F}_{\left\langle jk \right\rangle }}\left( t_{0}^{\left( j \right) } \right) {{e}^{-{{\Delta }_{j}}\left( t-t_{0}^{\left( j \right) } \right) }}, \end{aligned} \end{aligned}$$

where ${{\mathcal A}}_{j} \left( t,t_{0}^{\left( j\right) } \right) =e^{-\Delta _{j} \left( t-t_{0}^{\left( j\right) } \right) }$ is the survival amplitude of ${\left| \varphi _{N,jk}^{*} \left( t\right) \right\rangle }$ such that the target state is initialized at $t_{0}^{\left( j\right) }$, $F_{\left\langle jk\right\rangle } \left( t_{0}^{\left( j\right) } \right)$ is the initial target function value at $t_{0}^{\left( j\right) }$, while $\left\langle jl\right\rangle$ refer to the neighboring entangled connections of *j*, $l=1,\ldots ,\left( \Gamma _{j} +2\right) -1,l\ne k$, while $G_{l} \left( s\right)$ is a control parameter^148^, defined as

126 $$\begin{aligned} {{G}_{l}}\left( s \right) =\left\{ \begin{matrix} 0,\text {if }s<t_{0}^{\left( l \right) } \\ 1,\text {if }s\ge t_{0}^{\left( l \right) } \\ \end{matrix} \right. , \end{aligned}$$

where $$s\le T$$.

Using (124), the $\mu _{N} \left( t\right) =\left( \mu _{1} \left( t\right) ,\ldots ,\mu _{D} \left( t\right) \right) ^{T}$ cumulated target state intensity of *N* can be defined as

127 $$\begin{aligned} \mu _{N} \left( t\right) =\Lambda _{N} \left( t\right) +\int \limits _{0}^{t}F_{N} \left( t_{0} \right) {{\mathcal A}}_{N} \left( t,s\right) dG_{N} \left( s\right) , \end{aligned}$$

where $\Lambda _{N} \left( t\right)$ is the vector of target state intensities of *N*

128 $$\begin{aligned} \Lambda _{N} \left( t\right) =\Lambda _{1} \left( t,t_{0}^{\left( 1\right) } \right) ,\ldots ,\Lambda _{D} \left( t,t_{0}^{\left( D\right) } \right) ^{T} , \end{aligned}$$

and

129 $$\begin{aligned} \begin{aligned} {{F}_{N}}\left( {{t}_{0}} \right) {{\mathcal {A}}_{N}}\left( t,s \right)&={{\left[ {{F}_{\left\langle jl \right\rangle }}\left( t_{0}^{\left( l \right) } \right) {{\mathcal {A}}_{l}}\left( t,s \right) \right] }_{j,l=1,\ldots ,l={{\Gamma }_{j}}+1,l\ne k}} \\&={{\left[ {{F}_{\left\langle jl \right\rangle }}\left( t_{0}^{\left( l \right) } \right) {{e}^{-{{\Delta }_{l}}\left( t-s \right) }} \right] }_{j,l=1,\ldots ,l={{\Gamma }_{j}}+1,l\ne k}}, \end{aligned} \end{aligned}$$

while $G_{N} \left( s\right)$ is a vector of control parameters

130 $$\begin{aligned} G_{N} \left( s\right) =\left( G_{1} \left( t\right) ,\ldots ,G_{D} \left( t\right) \right) ^{T} . \end{aligned}$$

At that point, our aim is to reveal the impacts of a $M\left[ m_{b} \right]$ local measurement (performed in the ${{\mathcal L}}_{D}$ download phase) on the $\mu _{N} \left( t\right)$ cumulated target function intensity (127), i.e., to describe the impact of a local measurement and the localization process on the global entangled network structure.

Let us assume that a $M\left[ m_{b} \right]$ measurement is performed on a qubit *j* of $\left\langle jk\right\rangle$ at $\tau ^{\left( j\right) } \in \left[ 0,T\right]$, denoted by $M\left( \tau ^{\left( j\right) } \right) \left[ m_{b} \right]$. Then, let $\mu _{N} \left( t,\tau ^{\left( j\right) } \right)$ refer to the resulting cumulated target state intensity of *N*, evaluated as

131 $$\begin{aligned} \begin{aligned} {{\mu }_{N}}\left( t,{{\tau }^{\left( j \right) }} \right)&=\left( {{\Lambda }_{N}}\left( t \right) +\int \limits _{0}^{{{\tau }^{\left( j \right) }}}{{{F}_{N}}\left( {{t}_{0}} \right) {{\mathcal {A}}_{N}}\left( t,s \right) d{{G}_{N}}\left( s \right) } \right) \circ {{h}_{N}}\left( j \right) \circ D\left( B \right) \\&+\int \limits _{{{\tau }^{\left( j \right) }}}^{t}{\left( {{F}_{N}}\left( {{t}_{0}} \right) {{\mathcal {A}}_{N}}\left( t,s \right) d{{G}_{N}}\left( s \right) \right) ,} \end{aligned} \end{aligned}$$

where $\circ$ denotes element-wise product, $h_{N} \left(j\right)$ is a vector with indicators $h_{\left\langle xy\right\rangle }$ to identify the localized entangled connections of *N* that are entangled with qubit *j*, as

132 $$\begin{aligned} {{h}_{\left\langle xy \right\rangle }}=\left\{ \begin{matrix} 0,\text {if }x=j \\ 1,\text {otherwise} \\ \end{matrix} \right. , \end{aligned}$$

where $h_{\left\langle xy\right\rangle }$ is an indicator associated to $\left\langle xy\right\rangle$. Let $G_{N} \left( s,h\left( N\right) \right)$ be defined as^148–150^

133 $$\begin{aligned} G_{N} \left( s,h\left( N\right) \right) =G_{N}^{t\le \tau ^{\left( j\right) } } \left( s,h\left( N\right) \right) +G_{N}^{t>\tau ^{\left( j\right) } } \left( s,h\left( N\right) \right) , \end{aligned}$$

while $G_{N}^{t\le \tau ^{\left( j\right) } } \left( s,h\left( N\right) \right)$ is an indicator for $t\le \tau ^{\left( j\right) }$ (i.e., $G_{N}^{t\le \tau ^{\left( j\right) } } \left( s,h\left( N\right) \right)$ indicates the target state intensity before the localization in the intermediate node where the measurement is performed), while $G_{N}^{t>\tau ^{\left( j\right) } } \left( s,h\left( N\right) \right)$ is set for $t>\tau ^{\left( j\right) }$ (i.e., $G_{N}^{t>\tau ^{\left( j\right) } } \left( s,h\left( N\right) \right)$ indicates the target state intensity after the localization onto the receiver node), and $D\left( B\right) =\left( B_{1} ,\ldots ,B_{n} \right) ^{T}$ is a vector of *n* receivers for the localization procedure of target state intensity in the ${{\mathcal L}}_{D}$ downloading procedure, as

134 $$\begin{aligned} {{B}_{i}}=\left\{ \begin{matrix} 1,\text {if }j\in \mathcal {P}\left( {{A}_{i}}\rightarrow {{B}_{i}} \right) \\ 0,\text {otherwise} \\ \end{matrix} \right. , \end{aligned}$$

thus, if *j* belongs to the computational path $${\mathcal {P}}\left( A_{i} \rightarrow B_{i} \right) $$ then $$B_{i} $$ is a target node in $${{\mathcal L}}_{D} $$.

As follows, the $\mu _{N} \left( t,\tau ^{\left( j\right) } \right)$ cumulated target state intensity of the global entangled structure can be decomposed into a sum of target state intensities before measurement $M\left( \tau ^{\left( j\right) } \right) \left[ m_{b} \right]$ in the intermediate nodes, and after measurement in the target node. As a corollary of the $M\left( \tau ^{\left( j\right) } \right) \left[ m_{b} \right]$ measurement on *j*, for any $t\le \tau ^{\left( j\right) }$, the target state intensities of connections entangled with *j* vanish from the cumulated target state intensity in the intermediate nodes.

As the measurement on *j* is performed in the intermediate node, we focus to Bob $$B_{i} $$, to evaluate the target state intensity on his localized system state. As follows, at Bob $$B_{i} $$, the target state intensity of the localized system is as

135 $$\begin{aligned} \begin{aligned} {{\mu }_{{{B}_{i}}}}\left( t,{{\tau }^{\left( j \right) }} \right)&={{\Lambda }_{{{B}_{i}}}}\left( t \right) +\int \limits _{0}^{{{\tau }^{\left( j \right) }}}{{{F}_{{{B}_{i}}}}\left( {{t}_{0}} \right) \left( t \right) {{\mathcal {A}}_{{{B}_{i}}}}\left( t,s \right) dG_{{{B}_{i}}}^{t>{{\tau }^{\left( j \right) }}}\left( s,h\left( N \right) \right) } \\&=F_{\left\langle jk \right\rangle }^{{{B}_{i}}}\left( t_{0}^{\left( j \right) } \right) {{e}^{-{{\Delta }_{j}}\left( t-t_{0}^{\left( j \right) } \right) }}+\sum \limits _{l=1,l\ne k}^{{{\Gamma }_{j}}+1}{\int \limits _{0}^{{{\tau }^{\left( j \right) }}}{F_{\left\langle jl \right\rangle }^{{{B}_{i}}}\left( t_{0}^{\left( l \right) } \right) {{e}^{-{{\Delta }_{l}}\left( t-s \right) }}}}dG_{{{B}_{i}}}^{t>{{\tau }^{\left( j \right) }}}\left( s,h\left( N \right) \right) , \end{aligned} \end{aligned}$$

where index $$B_{i} $$ refers to the localized terms at Bob $$B_{i} $$.

The remaining, non-localized target function intensity belongs to the entangled connections in the intermediate network is evaluated as

136 $$\begin{aligned} \begin{aligned} {{\mu }_{N}}\left( {{\tau }^{\left( j \right) }},t \right) =&{{\Lambda }_{N}}\left( t \right) +\int \limits _{0}^{{{\tau }^{\left( j \right) }}}{{{F}_{N}}\left( {{t}_{0}} \right) {{\mathcal {A}}_{N}}\left( t,s \right) dG_{N}^{t\le {{\tau }^{\left( j \right) }}}\left( s,h\left( N \right) \right) } \\&+\int \limits _{{{\tau }^{\left( j \right) }}}^{t}{\left( {{F}_{N}}\left( {{t}_{0}} \right) {{\mathcal {A}}_{N}}\left( t,s \right) dG_{N}^{t>{{\tau }^{\left( j \right) }}}\left( s,h\left( N \right) \right) \right) ,} \end{aligned} \end{aligned}$$

therefore the target state intensities evolves further in the intermediate network, where the term $h_{N} \left( j\right)$ indicates that the entangled connections that affected by the measurement are vanished out from the cumulated intensity value.

Utilizing the framework of^148–150^, (131) can be rewritten in a closed-form as

137 $$\begin{aligned} \begin{aligned} {{\mu }_{N}}\left( t,{{\tau }^{\left( j \right) }} \right) =&\left( {{\nu }_{{{B}_{i}}}}\left( t \right) {{\Lambda }_{{{B}_{i}}}}\left( t \right) \right) \\&+\left( {{\Lambda }_{N}}\left( t \right) +\zeta \left( t-{{\tau }^{\left( j \right) }} \right) {{F}_{N}}\left( {{t}_{0}} \right) \right) \left( {{h}_{N}}\left( j \right) \circ y\left( {{\tau }^{\left( j \right) }} \right) \right) , \end{aligned} \end{aligned}$$

where

138 $$\begin{aligned} \zeta \left( t-\tau ^{\left( j\right) } \right) =e^{\left( F_{N} \left( t_{0} \right) -\Delta _{N} I\right) \left( t-\tau ^{\left( j\right) } \right) } , \end{aligned}$$

where $\Delta _{N}$ is a vector of decay rates of the entangled connections of *N*, $y\left( \tau ^{\left( j\right) } \right)$ is

139 $$\begin{aligned} y\left( \tau ^{\left( j\right) } \right) =\int \limits _{0}^{\tau ^{\left( j\right) } }e^{\Delta _{N} \left( \tau ^{\left( j\right) } -s\right) } dG_{N} \left( s\right) , \end{aligned}$$

while $\nu _{B_{i} } \left( t\right)$ is a matrix function^148,149^ associated to Bob’s localized system, as

140 $$\begin{aligned} \begin{aligned} {{\nu }_{{{B}_{i}}}}\left( t \right)&=I+\int \limits _{0}^{{{\tau }^{\left( j \right) }}}{{{F}_{{{B}_{i}}}}\left( {{t}_{0}} \right) {{\mathcal {A}}_{{{B}_{i}}}}\left( t,s \right) {{\nu }_{{{B}_{i}}}}\left( s \right) ds} \\&=I+{{F}_{{{B}_{i}}}}\left( {{t}_{0}} \right) {{\left( {{F}_{{{B}_{i}}}}\left( {{t}_{0}} \right) -{{\Delta }_{{{B}_{i}}}}I \right) }^{-1}}\left( {{e}^{\left( {{F}_{{{B}_{i}}}}\left( {{t}_{0}} \right) -{{\Delta }_{{{B}_{i}}}}I \right) t}}-I \right) , \end{aligned} \end{aligned}$$

where $\Delta _{B_{i} }$ is a vector of decay rates of the localized entangled connections.

Since the target function values $F_{\left\langle jk\right\rangle } \left( t\right)$ are determined by ${\left| \varphi _{N,jk}^{*} \left( t\right) \right\rangle }$, it follows that at a target state decoherence the $F_{\left\langle jk\right\rangle } \left( t\right)$ target function values are therefore scalable via the $M\left[ m_{b} \right]$ measurement associated to the localization procedure of the ${{\mathcal L}}_{D}$ downloading in the intermediate nodes.

The proof is concluded here. $\square$

**Figure 6:**
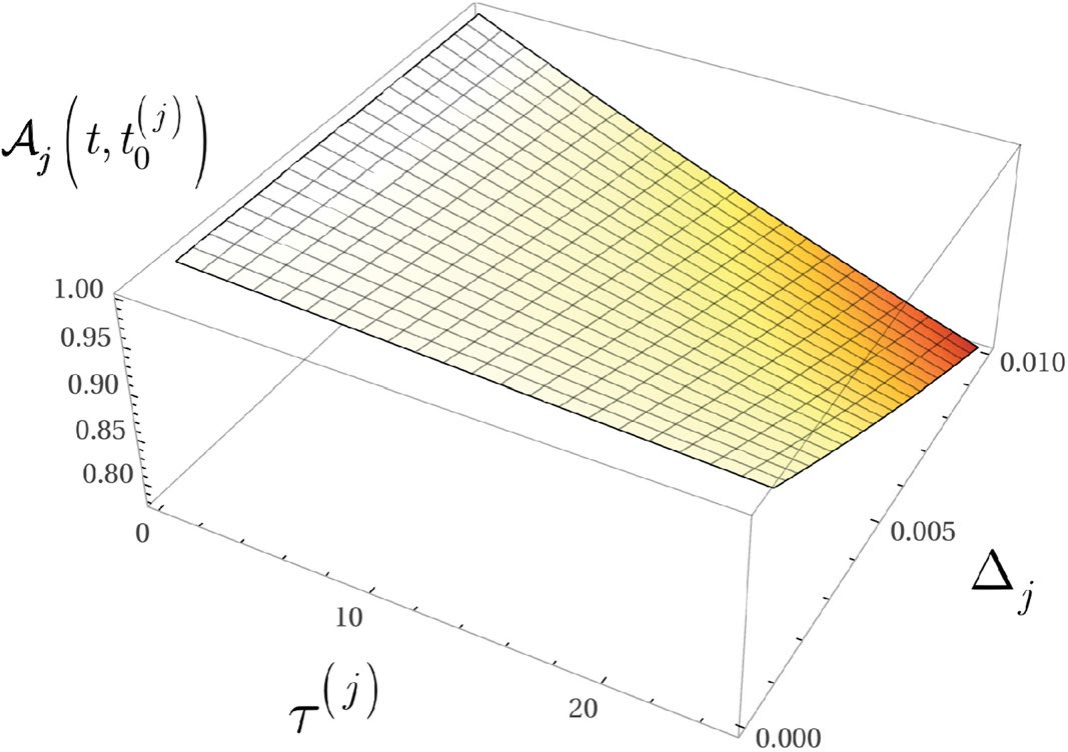
Scaling of the ${{\mathcal A}}_{j} \left( t,t_{0}^{\left( j\right) } \right)$ survival amplitude of the $\Lambda _{B_{i} } \left( t\right)$ target function intensity, $\Delta _{j} \in \left[ 10^{-3} ,10^{-2} \right]$, $\tau ^{\left( j\right) } \in \left[ 0,25\right]$, $t_{0}^{\left( j\right) } =0$.

The scaled ${{\mathcal A}}_{j} \left( t,t_{0}^{\left( j\right) } \right)$ survival amplitude of the $\Lambda _{B_{i} } \left( t\right)$ target function intensity of a given $\left\langle jk\right\rangle$ at different $\tau ^{\left( j\right) }$ measurement delays and $\Delta _{j}$ decay rates are depicted in Fig. 6.

### Scaled computational cost

#### **Lemma 2**

(Cost of target function evaluation). *The* $f_{C} \left( F_{\langle jk \rangle }\right)$ *computational cost associated to a given* $F_{\langle jk \rangle }$ *is the total application time of the local unitaries. The cost function is scalable via* $\Gamma _{j}$ *in a multipartite entanglement system*.

#### *Proof*

Let ${\mathcal {P}}\left( A\rightarrow B\right)$ be a computational path in *N* with *L* nodes and $\left( L-1\right)$ entangled connections. Then, for a given $\left\langle jk\right\rangle \in N$, let $\beta _{j}^{*}$, $\beta _{k}^{*}$ and $\gamma _{jk}^{*}$ refer to the gate parameters set to maximize the target function $F_{\langle jk \rangle }$, set via (109) and (110).

The $f_{C} \left( F_{{\mathcal {P}}\left( A\rightarrow B\right) } \right)$ computational cost of the maximization of target function $F_{{\mathcal {P}}\left( A\rightarrow B\right) }$ is defined as

141 $$\begin{aligned} f_{C} \left( F_{{\mathcal {P}}\left( A\rightarrow B\right) } \right) =\sum _{\left\langle jk\right\rangle \in {\mathcal {P}}\left( A\rightarrow B\right) }f_{C} \left( F_{\langle jk \rangle }\right) , \end{aligned}$$

where $f_{C} \left( F_{\langle jk \rangle }\right)$ is the computational cost associated to a given $F_{\langle jk \rangle }$ of an entangled connection $\left\langle jk\right\rangle$, as

142 $$\begin{aligned} f_{C} \left( F_{\langle jk \rangle }\right) =\beta _{j}^{*} +\beta _{k}^{*} +\gamma _{jk}^{*} ={\textstyle \frac{\pi }{4}} +{\textstyle \frac{1}{2}} \cos ^{-1} \left( {\textstyle \frac{\Gamma _{j} -1}{\Gamma _{j} +1}} \right) . \end{aligned}$$

that measures the computational cost as the total application time of the local unitaries.

As follows, (141) depends only on $\Gamma _{j}$, thus the scaling coefficient of the computational cost is $\Gamma _{j}$.

The $S_{R} \left( f_{C} \left( F_{\langle jk \rangle }\right) \right)$ series representation of (142) for $\left| {\Gamma _{j} \bigg / \left( 1+\Gamma _{j} \right) } \right| <1$, is

143 $$\begin{aligned} \begin{aligned} {{S}_{R}}\left( {{f}_{C}}\left( F_{\langle jk \rangle } \right) \right)&=\tfrac{1}{4}\left( 2{{\cos }^{-1}}\left( \tfrac{{{\Gamma }_{j}}-1}{{{\Gamma }_{j}}+1} \right) +\pi \right) \\&=\tfrac{3\pi }{4}-\sqrt{\tfrac{{{\Gamma }_{j}}}{{{\Gamma }_{j}}+1}}\sum \limits _{k=0}^{\infty }{\frac{\left( \tfrac{1}{2} \right) {{\left( \tfrac{{{\Gamma }_{j}}}{{{\Gamma }_{j}}+1} \right) }^{k}}}{2kk!+k!},} \end{aligned} \end{aligned}$$

while the $S_{E} \left( f_{C} \left( F_{\langle jk \rangle }\right) \right)$ series expansion of (142) at $\Gamma _{j} =\infty$ is as

144 $$\begin{aligned} \begin{aligned} S\left( {{f}_{C}}\left( F_{\langle jk \rangle } \right) \right) =&\tfrac{\pi }{4}+\sqrt{\tfrac{1}{{{\Gamma }_{j}}}}-\tfrac{1}{3}{{\left( \tfrac{1}{{{\Gamma }_{j}}} \right) }^{3/2}}+\tfrac{1}{5}{{\left( \tfrac{1}{{{\Gamma }_{j}}} \right) }^{5/2}} \\&-\tfrac{1}{7}{{\left( \tfrac{1}{{{\Gamma }_{j}}} \right) }^{7/2}}+\tfrac{1}{9}{{\left( \tfrac{1}{{{\Gamma }_{j}}} \right) }^{9/2}}+\mathcal {O}\left( {{\left( \tfrac{1}{{{\Gamma }_{j}}} \right) }^{5}} \right) . \end{aligned} \end{aligned}$$

The $f_{C} \left( N\right)$ total computational cost of *N* at *n* computational paths, is therefore

145 $$\begin{aligned} f_{C} \left( N\right) =\sum _{q=1}^{n}f_{C} \left( F_{{\mathcal {P}}_{q} } \right) =\sum _{q=1}^{n}\sum _{\left\langle jk\right\rangle \in {\mathcal {P}}_{q} }f_{C} \left( F_{\langle jk \rangle }\right) . \end{aligned}$$

$$\square $$

**Figure 7:**
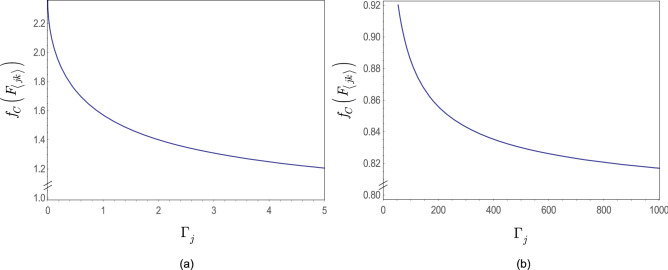
The cost function of $F_{\langle jk \rangle }$ scaled by (a) $\Gamma _{j} \in \left[ 0,5\right]$, and (b) $\Gamma _{j} \in \left[ 0,1000\right]$.

In Fig. 7, the scaled $f_{C} \left( F_{\langle jk \rangle }\right)$ cost function of $F_{\langle jk \rangle }$ is depicted.

## Conclusions

Here, we defined a scalable model of distributed gate-model quantum computation in near-term quantum systems. We evaluated the scaling attributes and the unitaries of a distributed system for solving optimization problems. We showed that the computational model is an extended correlation space. We studied how decoherence affects the distributed computational model and characterized a cost function. The proposed results are applicable in different scenarios of experimental gate-model quantum computations.

## Supplementary Information

Supplementary information.

## Data Availability

This work does not have any experimental data.
