# Supplementary material for: Scalable distributed gate-model quantum computers
Source: Sci Rep. 2021 Feb 26;11:5172. doi: 10.1038/s41598-020-76728-5 (PMC7910494; doi:10.1038/s41598-020-76728-5)
Supplement: Supplementary file 1 — Supplementary information. [file 41598_2020_76728_MOESM1_ESM.pdf]

Scalable Distributed Gate-Model Quantum Computers

Laszlo Gyongyosi[1,2,3,*] **and Sandor Imre**[2]

[1]School of Electronics and Computer Science, University of Southampton, Southampton, SO17 1BJ, UK
[2]Department of Networked Systems and Services, Budapest University of Technology and Economics, Budapest, H-1117 Hungary
[3]MTA-BME Information Systems Research Group, Hungarian Academy of Sciences, Budapest, H-1051 Hungary
[*]gyongyosi@hit.bme.hu

ABSTRACT

A scalable model for a distributed quantum computation is a challenging problem due to the complexity of the problem space provided by the diversity of possible quantum systems, from small-scale quantum devices to large-scale quantum computers. Here, we define a model of scalable distributed gate-model quantum computation in near-term quantum systems of the NISQ (noisy intermediate scale quantum) technology era. We prove that the proposed architecture can maximize an objective function of a computational problem in a distributed manner. We study the impacts of decoherence on distributed objective function evaluation.

A Appendix

A.1 Notations

The notations of the manuscript are summarized in Table A.1.

Table A.1. Summary of notations.

Notation	*Description*
N	A scalable distributed quantum system, $N=(V,E)$, where V is a set of nodes, E is a set of entangled connections. Refers to an arbitrary physical system (quantum device, quantum computer system, quantum network, quantum Internet system).
A_i	An i-th source user (quantum node) in the distributed quantum system.
B_i	An i-th target user (quantum node) in the distributed quantum system.
l	Level of entanglement. The quantum nodes share entanglement, the level of entanglement refers to the number of spanned intermediate nodes between a source and target node.
L	Number of nodes of a computational path in the distributed system.
n	Number of computational paths in the distributed system.
nL	Total number of quantum nodes in the distributed quantum system at n computational paths.
s,m,l	Small, medium and large scale quantum system.
X	Pauli X operator.
Z	Pauli Z operator.
Y	Pauli Y operator.
V_x	A quantum node from the distributed system.

V_{xy}	A node pair $V_{xy} = \left\{V_x, V_y\right\}$ of the distributed system connected by an arbitrary level of entanglement.
$\mathrm{L}_l\,(x,y)$	An l-level entangled connection between quantum nodes x and y, also denoted by $E\,(x,y)$.
$d\,(x,y)_{\mathrm{L}_l}$	Hop-distance at an L_l-level entangled connection between quantum nodes x and y, $d\,(x,y)_{\mathrm{L}_l} = 2^{l-1}$.
O_C	An oscillator with frequency f_C, $f_C = 1/t_C$, serves as a reference clock.
C	A cycle, with $t_C = 1/f_C$.
π_S	A time unit, defined as $\pi_S = xt_C$, where x is the number of C.
B_F	Entanglement throughput [Bell states per π_S].
$\lvert B_F \rvert$	Number of entangled states [Number of Bell states].
$E\,(x,y)$	An l-level entangled connection between quantum nodes x and y.
$B_F\,(E\,(x,y))$	Entanglement throughput of the entangled connection $E\,(x,y)$ [Bell states per π_S].
$\mathscr{P}\,(A \to B)$	A computational path of N, modeled as a set $V = \{V_1, \ldots, V_L\}$ of L quantum nodes, with a set $S = \{E_1, \ldots, E_{L-1}\}$ of $L-1$ entangled connections between the nodes, where E_j identifies an entangled connection between d-dimensional quantum states j and k (assumed to be qubit systems in a near-term system) in nodes V_x and V_y . A computational path contains L nodes, $L-1$ entangled connections and $2\,(L-1)$ entangled qubits.
V_{xy}	A node pair $V_{xy} = \left\{V_x, V_y\right\}$ with a shared l-level entangled connection.
$U\,(N)$	A unitary realized by the distributed system N.
$U\,(X_j, \beta_j)$	Local unitary, where X is the Pauli σ_x operator, while $\beta_j \in [0,\pi]$ is the gate parameter.
β_j	Gate parameter of the unitary $U\,(X_j, \beta_j)$, $\beta_j \in [0,\pi]$.
$U\left(Z_j Z_k, \gamma_{jk}\right)$	A distributed unitary $U\left(Z_j Z_k, \gamma_{jk}\right) = U\,(Z_j Z_k, \gamma_j)\,U\,(Z_j Z_k, \gamma_k)$, defined on qubits j and k using the l-level entangled connection $E_j = \langle jk \rangle$, where Z is the Pauli σ_z operator.
γ_{jk}	Gate parameter of the distributed unitary $U\left(Z_j Z_k, \gamma_{jk}\right)$, $\gamma_{jk} \in [0, 2\pi]$, defined as $\gamma_{jk} = \gamma_j + \gamma_k$, where $\gamma_j, \gamma_k \in [0,\pi]$ are the local gate parameters applied on qubits j and k.
γ_j, γ_k	Local gate parameters of unitary $U\left(Z_j Z_k, \gamma_{jk}\right)$, defined on qubits j and k, $\gamma_j, \gamma_k \in [0,\pi]$, $\gamma_j = \gamma_k = \frac{1}{2}\gamma_{jk}$.
$\langle jk \rangle$	An entangled connection between qubits j and k.
U_x^C	A local coupling unitary to connect qubits i and j from entangled connections $\langle (i-1)\,(i) \rangle$ and $\langle jk \rangle$ in V_x, as $U_x^C = \exp\left(-itH^{(i,j)}\right)$, where $H^{(i,j)}$ is a Hamiltonian.
U_{xy}	A unitary associated to a node pair $\left\{V_x, V_y\right\}$ connected by an l-level entanglement E_j in the distributed quantum system N.
U_x	Unitary associated to a node V_x, $x = 1, \ldots, L$, defined as $U_x = U\,(X_j, \beta_j)\,U\,(Z_j Z_k, \gamma_j)$.
U_y	Unitary of the neighbor node V_y (neighbor of V_x), defined as $U_y = U\,(X_k, \beta_k)\,U\,(Z_j Z_k, \gamma_k)$.

Γ_j	Number of remote entangled connections of qubit j, $n_1,\ldots,n_{\Gamma_j}$. Qubit j has entangled connection with k to formulate the entangled connection $\langle jk\rangle$, and also with Γ_j remote qubits which are not neighbors of qubit k.
$\vert +\rangle_i$	Input system of an i-th computational path, $\vert +\rangle_i = \frac{1}{\sqrt{2}}\left(\vert 0\rangle + \vert 1\rangle\right)$.
$\vert s\rangle$	The total input system of the distributed system N, distributed between n source users, defined as a product of σ_x eigenstates, as $\vert s\rangle = \vert +\rangle_1 \vert +\rangle_2 \ldots \vert +\rangle_n = \vert +\rangle^{\otimes n} = \frac{1}{\sqrt{2^n}}\sum_z \vert z\rangle$, where $\vert z\rangle$ is a computational basis state, z is an n-length string, $z = z_1 z_2 \ldots z_n$, where z_i identifies an i-th bit, $z_i \in \{-1,1\}$, and $\vert +\rangle_i$ is the input system of an i-th computational path $\mathscr{P}\left(A_i \to B_i\right)$.
$\vert \varphi^*\rangle$	Output of a computational path $\mathscr{P}\left(A \to B\right)$, $\vert \varphi^*\rangle = U_{\mathscr{P}(A\to B)}\vert +\rangle$, where $\vert +\rangle = \frac{1}{\sqrt{2}}\left(\vert 0\rangle + \vert 1\rangle\right)$.
$\vert \phi^*\rangle$	Output of the distributed system, $\vert \phi^*\rangle = U\left(N\right)\vert s\rangle$.
$C_{\mathscr{P}(A\to B)}$	Objective function of an arbitrary computational problem associated to computational path $\mathscr{P}\left(A \to B\right)$ in the distributed system N.
ζ_{E_j}	Contribution of an l-level E_j entangled connection between qubits j and k in target function $F_{\mathscr{P}(A\to B)}$, defined as $\zeta_{E_j} = \left(\sin\left(2\beta_j + 2\beta_k\right)\right)\sin\gamma_{jk}\prod_{k=1}^{\Gamma_j+1}\cos\gamma_{jk}$, where Γ_j is the number remote entangled qubits of j such that not neighbors of qubit k, while β_j, β_k and γ_{jk} are the gate parameters of unitaries.
$F_{\mathscr{P}(A\to B)}$	Target function of a computational path $\mathscr{P}\left(A \to B\right)$, defined as $F_{\mathscr{P}(A\to B)} = \max_{\forall\theta}\left\langle \varphi^*\right\vert C_{\mathscr{P}(A\to B)}\left\vert \varphi^*\right\rangle = \frac{1}{2}\sum_{j=1}^{L-1}\zeta_{E_j}$,
$U(\vec{\theta}_i)$	Unitary associated to an i-th path $\mathscr{P}\left(A_i \to B_i\right)$.
$U\left(N\right)$	The unitary realized by the distributed system N, $U\left(N\right) = \prod_{j\in N} U\left(X_j,\beta_j\right)\prod_{\langle jk\rangle\in N} U\left(Z_j Z_k,\gamma_{jk}\right) = U\left(\vec{\theta}_n\right)U\left(\vec{\theta}_{n-1}\right)\ldots U\left(\vec{\theta}_1\right)$, where $U(\vec{\theta}_i)$ refer to the unitary associated to an i-th path $\mathscr{P}\left(A_i \to B_i\right)$.
z	A bitstring for the evaluation of the $C\left(z\right)$ objective function of a computational problem fed into the distributed system.
$M\left[m_b\right]$	A local measurement in the intermediate network in a base $m_b \in \{m_0, m_1\}$.
M	Distributed measurement in the n receiver nodes $B_1,\ldots,B_n$ to obtain output string z.
$\mathscr{L}_U$	Upload procedure, an information delocalization method, in which a source system is uploaded (correlated) by a source node onto the network state.
$\mathscr{L}_D$	Download procedure, an information localization procedure, in which the uploaded and transformed information (transformed by the local unitaries of the intermediate nodes) is localized (decorrelated from the network state via local measurements) into a particular target node from the network state of intermediate nodes.
$\mathscr{M}_B$	Bell measurement, applied in the source nodes for the uploading. The measurement uses the first particle (subsystem A of the first Bell pair AB of the network state) of the network state as an auxiliary system.
$\vert \Phi\rangle_i$	Network state of computational path $\mathscr{P}\left(A_i \to B_i\right)$, defined as $\vert \Phi\rangle_i = U(\vec{\theta}_i)\frac{1}{\sqrt{2}}\left(\vert 0\rangle_{aux}\left(\vert 0\rangle\right)_2^{2(L-1)} + \vert 1\rangle_{aux}\left(\vert 1\rangle\right)_2^{2(L-1)}\right)$,where sub-index 1 identifies the first particle of $\vert \Phi\rangle_i$ of $\mathscr{P}\left(A_i \to B_i\right)$ maximally entangled with the remaining $2\left(L-1\right)$ qubits of the chain of $\mathscr{P}\left(A_i \to B_i\right)$.

$\|\Phi\rangle_1^n$	Distributed network state formulated via n computational paths $\mathscr{P}(A_1 \to B_1),\ldots,\mathscr{P}(A_n \to B_n)$, as $\|\Phi\rangle_1^n = U(N)\frac{1}{\sqrt{2}}\left((\|00\rangle)_1^{n2(L-1)} + (\|11\rangle)_1^{n2(L-1)}\right) = U(N)\frac{1}{\sqrt{2}}\left(\|0\rangle_1^n(\|0\rangle)_{n+1}^{n2(L-1)} + \|1\rangle_1^n(\|1\rangle)_{n+1}^{n2(L-1)}\right)$, where indices $1,\ldots,n$ identify the auxiliary systems used for the uploading procedure in the n nodes.
$\|aux\rangle$	An auxiliary qubit system, $\mathscr{H}_{aux} = \mathbb{C}^2$, refers to the first particle of the network state. It is used in the Bell measurement of the uploading procedure.
$(\|L\rangle)_2^{2(L-1)}$	An $2(L-1)$-qubit length system, $\mathscr{H}_L = \mathbb{C}^{2^{\otimes 2(L-1)}}$, that identifies the network state $\|\Phi\rangle_i$ of a computational path $\mathscr{P}(A_i \to B_i)$, formulating orthogonal states as $(\|L\rangle)_2^{2(L-1)} = \left\{\|0\rangle_2^{2(L-1)}, \|1\rangle_2^{2(L-1)}\right\}$.
$\|\Phi'\rangle_i$	Post-measurement network state of an i-th computational path $\mathscr{P}(A_i \to B_i)$ (after the measurements are performed the intermediate nodes for the downloading).
$\|\Phi'\rangle_1^n$	Post-measurement network state of the distributed network N (after the measurements are performed the intermediate nodes for the downloading).
m_b	Bases for the local measurement $M[m_b]$ in the intermediate nodes, $m_b \in \{m_0, m_1\}$.
ς	Measurement parameter, $\varsigma \in [0,\pi]$, defined for measurement $M[m_b]$, $M[m_0] = \|\psi_0\rangle\langle 0\|$ and $M[m_1] = \|\psi_1\rangle\langle 1\|$, where $\|\psi_0\rangle = \cos\frac{\varsigma}{2}\|0\rangle + e^{i\alpha}\sin\frac{\varsigma}{2}\|1\rangle$, $\|\psi_1\rangle = \sin\frac{\varsigma}{2}\|0\rangle - e^{i\alpha}\cos\frac{\varsigma}{2}\|1\rangle$.
$C_{jk}(z)$	An objective function component evaluated for entangled connection $\langle jk\rangle \in N$, as $C_{jk}(z) = \frac{1}{2}(1 - z_j z_k)$, where z is an n-length input bitstring, $z = z_1 z_2 \ldots z_n$, and z_i identifies an i-th bit, $z_i \in \{-1, 1\}$.
$C(z)$	At a particular physical entangled connection topology in N, the objective function C can be written as $C(z) = \sum_{\langle jk\rangle \in N} C_{jk}(z)$.
$\|z\rangle$	A computational basis state, $\|z\rangle = \|z_1 z_2 \ldots z_n\rangle$.
$\left\|\varphi^*_{N,jk}\right\rangle$	A target state in the distributed system, defined as $\left\|\varphi^*_{N,jk}\right\rangle = \|\gamma_{jk}, \beta_k, \beta_j, C_{jk}\rangle = U(B,\beta_j)U(B,\beta_k)U(C_{jk}(z), \gamma_{jk})\|s\rangle$.
$F_{\langle jk\rangle}$	A target function defined for particular entangled connection $\langle jk\rangle$ of N, as $F_{\langle jk\rangle} = \max\limits_{\forall\theta}\left(\left(-\frac{1}{2}\right)\left\langle \varphi^*_{N,jk} \middle\| Z_j Z_k \middle\| \varphi^*_{N,jk}\right\rangle\right)$.
F	Target function defined for the distributed system N, as $F = \sum_{\langle jk\rangle \in N} F_{\langle jk\rangle} = \max\limits_{\forall\theta}\langle\varphi^* \mid C \| \varphi^*\rangle$.
$\mathscr{D}(N)$	A computational model of N, an extended correlation space.
$M[x_i]$	A 2×2 matrix, defined as $M[x_i] = \bar{c}_0^{(i)} M[0] + \bar{c}_1^{(i)} M[1]$.
$\|x_i\rangle$	A local state vector associated to node V_i, as $\|x_i\rangle = c_0^{(i)}\|0\rangle + c_1^{(i)}\|1\rangle$.
$\|A\rangle, \|B\rangle$	Input and output systems (boundary conditions in the extended correlation space), $d = 2$ dimensional vectors.
δ_i	Coefficient, set via the $\varsigma \in [0,\pi]$ measurement coefficient used in the definition of measurement operators as $\delta_i = \arg\left(\sin(\omega_i) + \cos(\omega_i)\exp\left(i\frac{\varsigma}{2}\right)\right)$, where ω_i identifies computational bases $\|b_{\omega_i}\rangle \in \{\|0_{\omega_i}\rangle, \|1_{\omega_i}\rangle\}$.
$D(\omega_i, \varsigma)$	A diagonal matrix.
$\mathscr{D}(\mathscr{P}(A \to B))$	The map of the physical computational path $\mathscr{P}(A \to B)$ onto the correlation space.

$S(x)$	A matrix in the extended correlation space, defined $S(x) = diag\left(e^{\frac{-ix}{2}}, e^{\frac{ix}{2}}\right)$.
W	A matrix in the extended correlation space, set as $W = \exp\left(i\pi \frac{X}{\mho}\right)$, where $\mho$ is a coefficient.
$F_{\mathscr{P}(A \to B)}$	Target function of the computational path $\mathscr{P}(A \to B)$.
$C_{\mathscr{P}(A \to B)}$	Objective function of the computational path $\mathscr{P}(A \to B)$.
$\left\lvert \varphi^{*}_{N,jk}(t) \right\rangle$	A time-dependent target state, target state of the distributed system at a time t.
$\mathscr{S}_{\varphi^{*}_{N,jk}}(t)$	Time evolution of target state $\left\lvert \varphi^{*}_{N,jk}(t) \right\rangle$.
$\mathscr{A}(t)$	Survival amplitude, from the exponential decay law, $\mathscr{A}(t) = e^{-\Delta t}$, where Δ is the decay rate.
$\hat{U}(t, t_0)$	Time evolution operator generated by a Hamiltonian $\hat{H}$.
Δ	Decay rate, from the exponential decay law, $\mathscr{A}(t) = e^{-\Delta t}$,
$F_{\langle jk \rangle}(t)$	Target function $F_{\langle jk \rangle}$ at a given t.
$t_{\langle ij \rangle}(N)$	A vector of initialization time values of the target states of the distributed system.
$\left\lvert \varphi^{*}_{N,jk}(t_0) \right\rangle$	Target state at an initialization time $t_0^{(j)} \in [0, T]$, $j = 1, \ldots, D$, where $D = n(L-1)$.
Λ	Intensity of a target state $\left\lvert \varphi^{*}_{N,jk}(t) \right\rangle$.
$\mathscr{A}_j\left(t, t_0^{(j)}\right)$	Survival amplitude of the $\Lambda_{B_i}(t)$ target function intensity of a given $\langle jk \rangle$.
$\mathscr{A}_N(t)$	Vector of survival amplitudes associated to the D target states, $D = n(L-1)$.
$M\left(\tau^{(j)}\right)[m_b]$	A measurement $M[m_b]$ on qubit j of $\langle jk \rangle$ at a time $\tau^{(i)} \in [0, T]$ in N, $i = 1, \ldots, 2D$, defined as $M\left(\tau^{(j)}\right)[m_b] = \begin{cases} 1, \text{if } j \text{ is measured at } \tau^{(j)} \\ 0, \text{otherwise.} \end{cases}$
$\mu_j(t)$	Cumulated target state intensity.
$\Lambda_{\langle jk \rangle}\left(t, t_0^{(j)}\right)$	Intensity of a target state $\left\lvert \varphi^{*}_{N,jk}(t) \right\rangle$ at a given t, the target state is initialized at $t_0^{(j)}$.
$\mathscr{A}_j\left(t, t_0^{(j)}\right)$	Survival amplitude of $\left\lvert \varphi^{*}_{N,jk}(t) \right\rangle$, at a target state initialization time $t_0^{(j)}$, $\mathscr{A}_j\left(t, t_0^{(j)}\right) = e^{-\Delta_j\left(t - t_0^{(j)}\right)}$.
$F_{\langle jk \rangle}\left(t_0^{(j)}\right)$	Target function value at $t_0^{(j)}$.
$G_l(s)$	A control parameter.
$\mu_N(t)$	Cumulated target state intensity of N, $\mu_N(t) = (\mu_1(t), \ldots, \mu_D(t))^T$.
$\Lambda_N(t)$	Vector of target state intensities of N, $\Lambda_N(t) = \Lambda_1\left(t, t_0^{(1)}\right), \ldots, \Lambda_D\left(t, t_0^{(D)}\right)^T$.
$G_N(s)$	Vector of control parameters, $G_N(s) = (G_1(t), \ldots, G_D(t))^T$.
$h_N(j)$	An indicator vector.
$h_{\langle xy \rangle}$	An indicator, associated to connection $\langle xy \rangle$.
$D(B)$	A vector of n receivers for the localization procedure of target state intensity in the $\mathscr{L}_D$ downloading procedure.
Δ_N	A vector of decay rates of the entangled connections of N.
$\zeta\left(t - \tau^{(j)}\right)$	A function in the target function scaling procedure.

$y\left(\tau^{(j)}\right)$	A function in the target function scaling procedure.
Δ_{B_i}	A vector of decay rates of the localized entangled connections.
$\nu_{B_i}(t)$	A matrix function, associated to the localized system of Bob.
$f_C\left(F_{\langle jk\rangle}\right)$	Computational cost associated to a given $F_{\langle jk\rangle}$.
$S_R\left(f_C\left(F_{\langle jk\rangle}\right)\right)$	A series representation of $f_C\left(F_{\langle jk\rangle}\right)$.
$S_E\left(f_C\left(F_{\langle jk\rangle}\right)\right)$	A series expansion of $f_C\left(F_{\langle jk\rangle}\right)$.
